# Central memory CD8^+^ T cells become CD69+ tissue-residents during viral skin infection independent of CD62L-mediated lymph node surveillance

**DOI:** 10.1371/journal.ppat.1007633

**Published:** 2019-03-15

**Authors:** Jossef F. Osborn, Samuel J. Hobbs, Jana L. Mooster, Tahsin N. Khan, Augustus M. Kilgore, Jake C. Harbour, Jeffrey C. Nolz

**Affiliations:** 1 Department of Molecular Microbiology & Immunology, Oregon Health & Science University, Portland, Oregon, United States of America; 2 Department of Cell, Developmental & Cancer Biology, Oregon Health & Science University, Portland, Oregon, United States of America; 3 Radiation Medicine, Oregon Health & Science University, Portland, Oregon, United States of America; University of Florida, UNITED STATES

## Abstract

Memory CD8^+^ T cells in the circulation rapidly infiltrate non-lymphoid tissues following infection and provide protective immunity in an antigen-specific manner. However, the subsequent fate of memory CD8^+^ T cells after entering non-lymphoid tissues such as the skin during a secondary infection is largely unknown. Furthermore, because expression of CD62L is often used to identify the central memory (T_CM_) CD8^+^ T cell subset, uncoupling the physical requirement for CD62L-mediated lymph node homing versus other functional attributes of T_CM_ CD8^+^ T cells remains unresolved. Here, we show that in contrast to naïve CD8^+^ T cells, memory CD8^+^ T cells traffic into the skin independent of CD62L-mediated lymph node re-activation and provide robust protective immunity against Vaccinia virus (VacV) infection. T_CM_, but not effector memory (T_EM_), CD8^+^ T cells differentiated into functional CD69+/CD103- tissue residents following viral clearance, which was also dependent on local recognition of antigen in the skin microenvironment. Finally, we found that memory CD8^+^ T cells expressed granzyme B after trafficking into the skin and utilized cytolysis to provide protective immunity against VacV infection. Collectively, these findings demonstrate that T_CM_ CD8^+^ T cells become cytolytic following rapid infiltration of the skin to protect against viral infection and subsequently differentiate into functional CD69+ tissue-residents.

## Author summary

Following vaccination or resolution of viral infection, three distinct populations of virus-specific memory CD8^+^ T cells are generated. Central memory cells (T_CM_) circulate between blood and lymph nodes and are able to rapidly enter non-lymphoid tissues in response to infection or tissue injury. In contrast, effector memory (T_EM_) are largely restricted to the blood and tissue-resident memory cells (T_RM_) are primarily confined within non-lymphoid tissues. In this study, we set out to interrogate the functionality and lineage relationship of these memory populations following a secondary viral skin infection. Circulating memory CD8^+^ T cells rapidly infiltrated the skin following viral infection and used cytolysis to provide robust protective immunity. T_CM_, but not T_EM_, trafficked into the skin and differentiated into functional T_RM_ following resolution of viral infection and this occurred independent of the ability of these cells to home into and survey peripheral lymph nodes. Altogether, our findings demonstrate that a defined lineage of circulating memory CD8^+^ T cells are the principle responders against viral skin infection and also the precursors to secondary T_RM_ CD8^+^ T cells that then form, which are important considerations for understanding host defense mechanisms against recurring viral infections and tissue-specific vaccine development.

## Introduction

Following acute infection or successful vaccination, antigen-specific CD8^+^ T cells that survive contraction differentiate into long-lived memory cells that provide protective immunity against re-infection. Memory CD8^+^ T cells exhibit a variety of functional characteristics (e.g. production of cytokines, cytolytic activity) that allow them to provide protective immunity against viruses and other intracellular pathogens [1, 2]. Another key feature of memory CD8^+^ T cells is their acquired ability to infiltrate and/or be retained in non-lymphoid tissues, whereas naïve CD8^+^ T cells are largely restricted to the circulation and secondary lymphoid organs [3, 4]. Memory CD8^+^ T cells can be broadly classified as those that are present in the circulation and those that remain in non-lymphoid tissues, now referred to as “tissue-resident” memory cells (T_RM_) [5]. Because of their location, often at environmental barriers, it is believed that pro-inflammatory T_RM_ CD8^+^ T cells are the cells most responsible for immediate protection against re-infections [6–10], prior to the trafficking of additional circulating memory CD8^+^ T cells into the site of infection to further promote pathogen clearance [11]. Understanding the mechanisms that control both the formation and protective features of these diverse memory T cell populations remains a challenge for rational vaccine design and development.

Both T_RM_ and memory CD8^+^ T cells in the circulation provide protective immunity against re-infections of the skin and other non-lymphoid tissues. Following T cell receptor (TCR) engagement, T_RM_ CD8^+^ T cells rapidly produce IFNγ that initiates a local inflammatory response and also promotes a general “anti-viral” state in the tissue microenvironment [12, 13]. Alternatively, in response to inflammatory cues, circulating memory CD8^+^ T cells rapidly enter non-lymphoid tissues and also provide significant protective immunity against viral skin infection [14, 15]. Memory CD8^+^ T cells in the circulation are often classified as being either central (T_CM_) or effector (T_EM_) memory based on the expression of receptors required for lymph node homing (i.e. CD62L and CCR7) [16]. We have recently demonstrated that T_CM_ CD8^+^ T cells are the primary memory cell subset that rapidly infiltrates the skin following VacV infection, whereas T_EM_ CD8^+^ T cells are mostly confined to the circulation [15]. Because CD62L is often used to identify the T_CM_ subset in mice, uncoupling the physical requirement for CD62L-mediated lymph node homing versus other functional attributes of T_CM_ CD8^+^ T cells during a secondary challenge has, for the most part, been correlative. Furthermore, the protective mechanisms used by circulating memory CD8^+^ T cells to control viral infection after trafficking into the skin are largely undefined.

The role that the tissue microenvironment plays in regulating the differentiation of T_RM_ CD8^+^ T cells remains controversial. In contrast to systemic viral infections such as lymphocytic choriomeningitis virus (LCMV), infection of the skin with herpes simplex virus-1 (HSV-1) or Vaccinia virus (VacV) activates naïve antigen-specific CD8^+^ T cells that subsequently traffic into and differentiate into T_RM_ almost exclusively at the site of infection [17–19], suggesting that local inflammation, at least in part, is critical for promoting the acquisition of the T_RM_ phenotype. Tissue-resident memory CD8^+^ T cells are often identified by expression or co-expression of CD69 and CD103 (α_E_ integrin) and both of these proteins contribute to the formation of T_RM_ in the skin [20, 21]. When paired with β_7_ integrin, CD103 becomes a receptor for E-cadherin and it has been proposed that this interaction is critical for retaining CD8^+^ T cells in the epidermis. An analysis of skin from healthy human subjects agrees with this model, as CD103-expressing CD8^+^ T cells are enriched in the epidermis compared to the cells that only express CD69 [22]. TGFβ-receptor signaling causes effector CD8^+^ T cells to express CD103, suggesting that the capacity to respond to TGF-β may be an important retention signal that facilitates T_RM_ differentiation in the skin and other E-cadherin-rich non-lymphoid tissues [20, 23]. In contrast, overexpression of genes that promote tissue egress including the transcription factor KLF2 or the sphingosine-1-phosphate receptor (S1P_1_; *S1PR1*) antagonizes T_RM_ development [24]. Collectively, these findings suggest that within tissue microenvironments, the ultimate fate of activated CD8^+^ T cells is highly dependent on the integration of conflicting signals that either promote or inhibit non-lymphoid tissue recruitment and retention. However, the signaling mechanisms within the tissue microenvironment and the cellular origins of either the CD69+ or CD69+/CD103+ T_RM_ subsets in the skin remain largely undefined.

In this study, we analyzed the functionality and lineage relationship of memory CD8^+^ T cell populations following a secondary viral skin infection. We found that circulating memory CD8^+^ T cells rapidly traffic into the skin following VacV infection independent of their ability to survey peripheral lymph nodes and provide significant protective immunity. In contrast to naïve CD8^+^ T cells, most circulating T_CM_ CD8^+^ T cells do not express CD103 in the skin microenvironment during the resolution of viral infection and only a subset ultimately became functional CD69+/CD103- T_RM_. Finally, after entering VacV-infected skin, circulating memory CD8^+^ T cells expressed granzyme B and required cytolytic function to provide protection against VacV infection. Altogether, this study demonstrates that T_CM_ CD8^+^ T cells will rapidly infiltrate non-lymphoid tissues in response to local inflammatory cues, accelerate viral clearance, and become secondary CD69+ T_RM_ that form following the resolution of viral skin infection, an important consideration for tissue-specific vaccine development.

## Results

### Circulating memory CD8^+^ T cells rapidly traffic into the skin and provide protective immunity against viral skin infection

Acute, systemic infection with LCMV-Armstrong causes antigen-specific T_RM_ CD8^+^ T cells to be seeded in a variety of non-lymphoid tissues (e.g. lung, gut, female reproductive tract) [23]. To determine if acute LCMV infection would also generate T_RM_ CD8^+^ T cells in the skin, naive P14 TCR-transgenic CD8^+^ T cells specific for LCMV GP_33-41_ were transferred into naïve B6 mice and infected with either LCMV by intraperitoneal (i.p.) injection or VacV expressing GP_33-41_ (VacV-GP33) by skin scarification. At 40 days post-infection, the distribution of memory P14 CD8^+^ T cells was analyzed in the skin, circulation, and lymphoid tissues (**S1A and S1B Fig**). As we have shown previously, infection of the skin with VacV generated both circulating memory CD8^+^ T cells as well as T_RM_ in the previously infected skin. In contrast, limited numbers of memory P14 CD8^+^ T cells could be isolated from the skin of mice infected with LCMV and these cells did not express the canonical T_RM_ markers CD69 or CD103 (**S1C and S1D Fig**). Thus, acute LCMV infection generates circulating memory CD8^+^ T cells, but does not generate T_RM_ CD8^+^ T cells in the skin.

**Fig 1:**
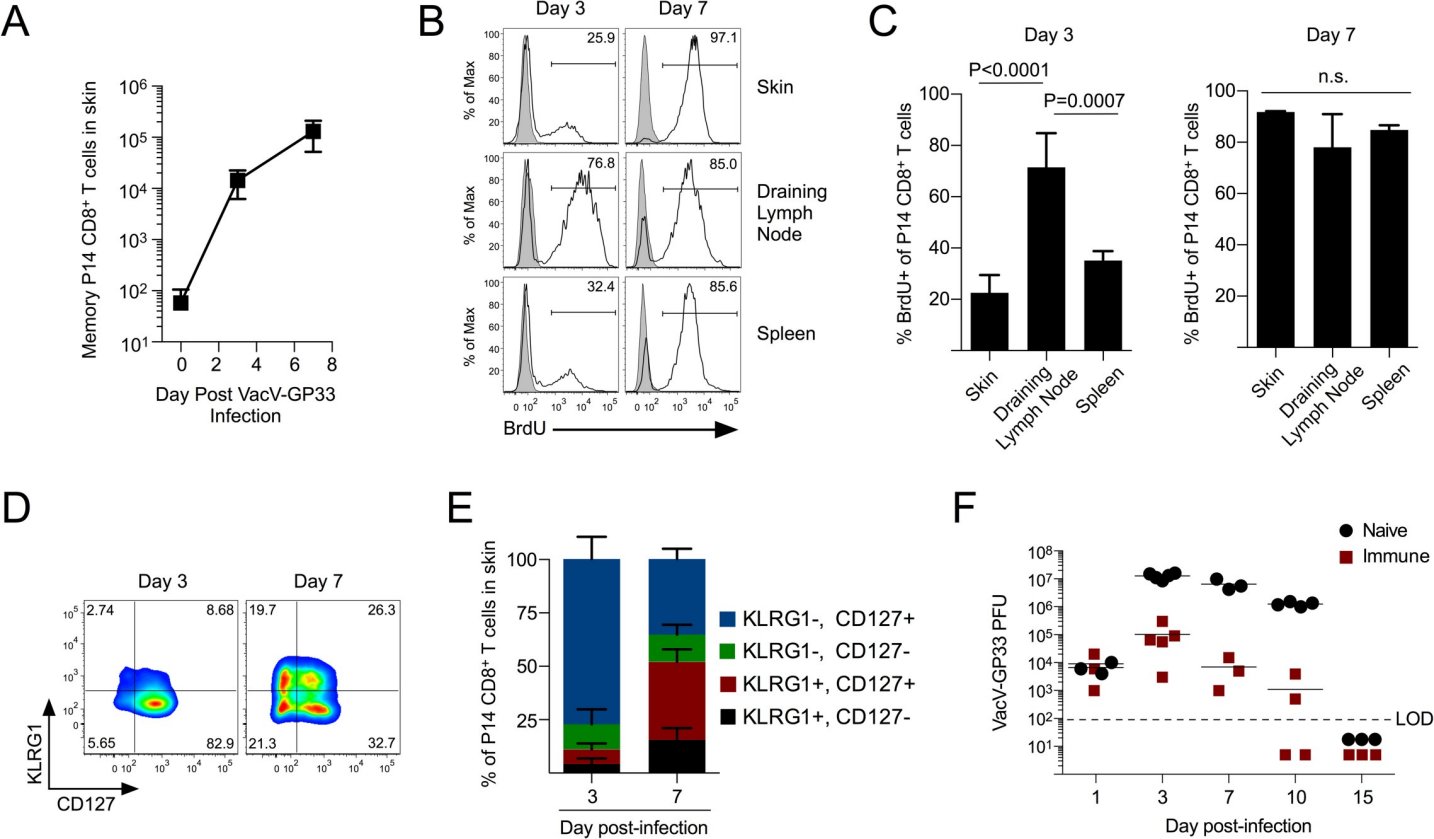
Antigen-specific memory CD8^+^ T cells rapidly traffic into VacV-infected skin and provide protective immunity. (A) Naïve P14 CD8^+^ T cells were transferred into B6 mice and infected with LCMV. At 75 days post LCMV-infection, mice were challenged with VacV-GP33 on the left ear skin and trafficking of memory P14 CD8^+^ T cells into the skin was quantified. (B) Proliferation of memory CD8^+^ T cells from (A) was analyzed in the indicated tissue by BrdU incorporation. (C) Quantification of (B). (D) Expression of CD127 and KLRG1 on memory P14 CD8^+^ T cells that trafficked in to the skin as shown in (A). (E) Quantification of (D). (F) Naïve and LCMV-immune mice were infected with 1 x 10^6^ PFU of VacV-GP33 on the left ear skin. On the indicated day post-infection, viral load was quantified in the infected skin. LOD (limit of detection) is indicated by the dashed line.

Because LCMV infection did not seed T_RM_ into the skin, we used the strategy of infecting LCMV-immune mice with VacV-GP33 to specifically monitor the antigen-specific memory CD8^+^ T cell recall response against viral skin infection. As we have shown previously [14, 15], circulating memory CD8^+^ T cells rapidly infiltrated the VacV-infected skin by day 3 post-infection, which was further increased by another ~10 fold on day 7 (**Fig 1A**). On day 3 post-infection, memory P14 CD8^+^ T cells had proliferated in the draining lymph node, but the majority of the cells that trafficked into VacV-infected skin had not proliferated and were CD127+/KLRG1- (**Fig 1B–1E**), suggesting that memory CD8^+^ T cells initially trafficked into the skin directly from the circulation and were not “re-primed” in the draining lymph node prior to infiltrating the skin microenvironment. In contrast, on day 7 post-infection, a variety of different populations of effector/memory CD8^+^ T cells were detected in the skin (based on expression of CD127 and KLRG1) and nearly all of the cells had proliferated. Antigen-specific memory CD8^+^ T cells reduced VacV burden in the skin as early as day 3 post-infection and began clearing the infection approximately 5 days earlier compared to naïve controls (**Fig 1F**). Thus, these data agree with the model that circulating memory CD8^+^ T cells respond to re-challenge to provide protective immunity in two phases [11]. Initially, memory CD8^+^ T cells directly infiltrate inflamed, non-lymphoid tissues to immediately control infection, which is followed by the subsequent recruitment of effector/memory CD8^+^ T cells that were re-activated in the draining lymph node.

### Circulating memory CD8^+^ T cells traffic into VacV-infected skin in a CD62L-independent fashion and become CD69+ after viral clearance

**Fig 2:**
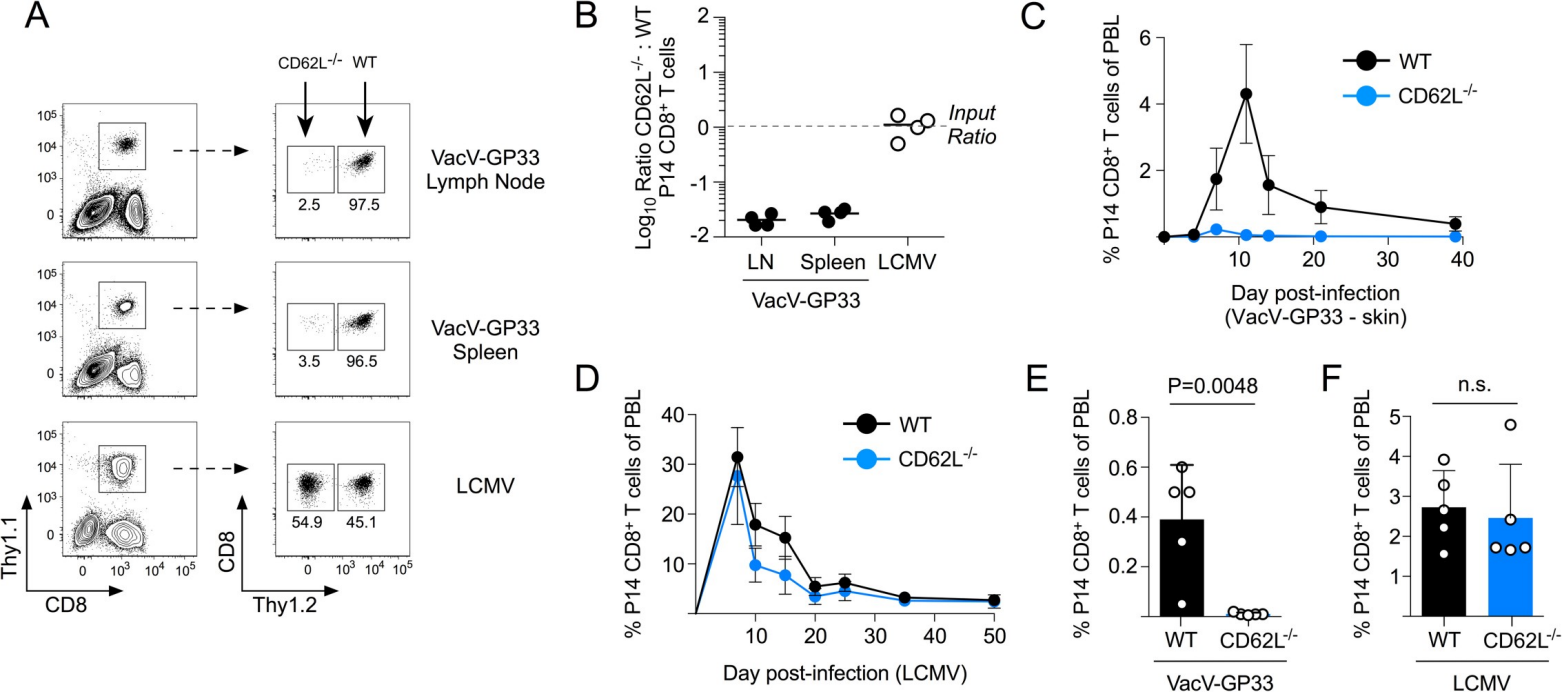
CD62L-dependent lymph node trafficking is required for naïve CD8^+^ T cells to become activated following VacV skin infection, but not systemic LCMV infection. (A) Equal numbers of WT (Thy1.1/1.2) and CD62L^-/-^ (Thy1.1/1.1) naïve P14 CD8^+^ T cells were transferred into B6 mice and infected with either VacV-GP33 by skin scarification or LCMV by i.p injection. Frequencies of the transferred P14 CD8^+^ T cells was evaluated on the peak day of expansion (day 10 for VacV-GP33, day 7 for LCMV). (B) Quantification of (A). (C) WT or CD62L^-/-^ naïve P14 CD8^+^ T cells were transferred into separate naïve B6 mice and infected with VacV-GP33 on the left ear skin. Expansion of the transferred T cells was then quantified. (D) Same as (C) except mice were infected with LCMV. (E, F) Quantification of circulating memory WT and CD62L^-/-^ P14 CD8^+^ T cells on day 40 post-infection.

CD62L is required for naïve T cells to adhere to and traffic across high endothelial venules and into peripheral lymph nodes [25, 26]. To determine whether CD62L was required for naïve CD8^+^ T cells to become activated during VacV skin infection, equal numbers of naïve WT (Thy1.1/1.2) and CD62L^-/-^ P14 CD8^+^ T cells (Thy1.1/1.1) were transferred into naïve B6 mice (Thy1.2/1.2) and infected with either VacV-GP33 on the skin or LCMV by i.p. injection. Following VacV-GP33 skin infection, expansion of WT, but not CD62L^-/-^ P14 CD8^+^ T cells was observed in both the draining lymph node and in the spleen, whereas both WT and CD62L^-/-^ naïve P14 CD8^+^ T cells expanded the same following systemic LCMV infection (**Fig 2A and 2B**). An extended longitudinal analysis of blood demonstrated that CD62L was necessary for antigen-specific naïve CD8^+^ T cell activation and expansion following VacV-GP33 skin infection, but was not required for the expansion, contraction and establishment of circulating antigen-specific memory CD8^+^ T cells following systemic LCMV infection (**Fig 2C–2F**). Thus, the capacity to traffic into lymph nodes is necessary for naïve CD8^+^ T cells to become activated during a peripheral skin infection, but is not required for their activation during systemic viral infection.

**Fig 3:**
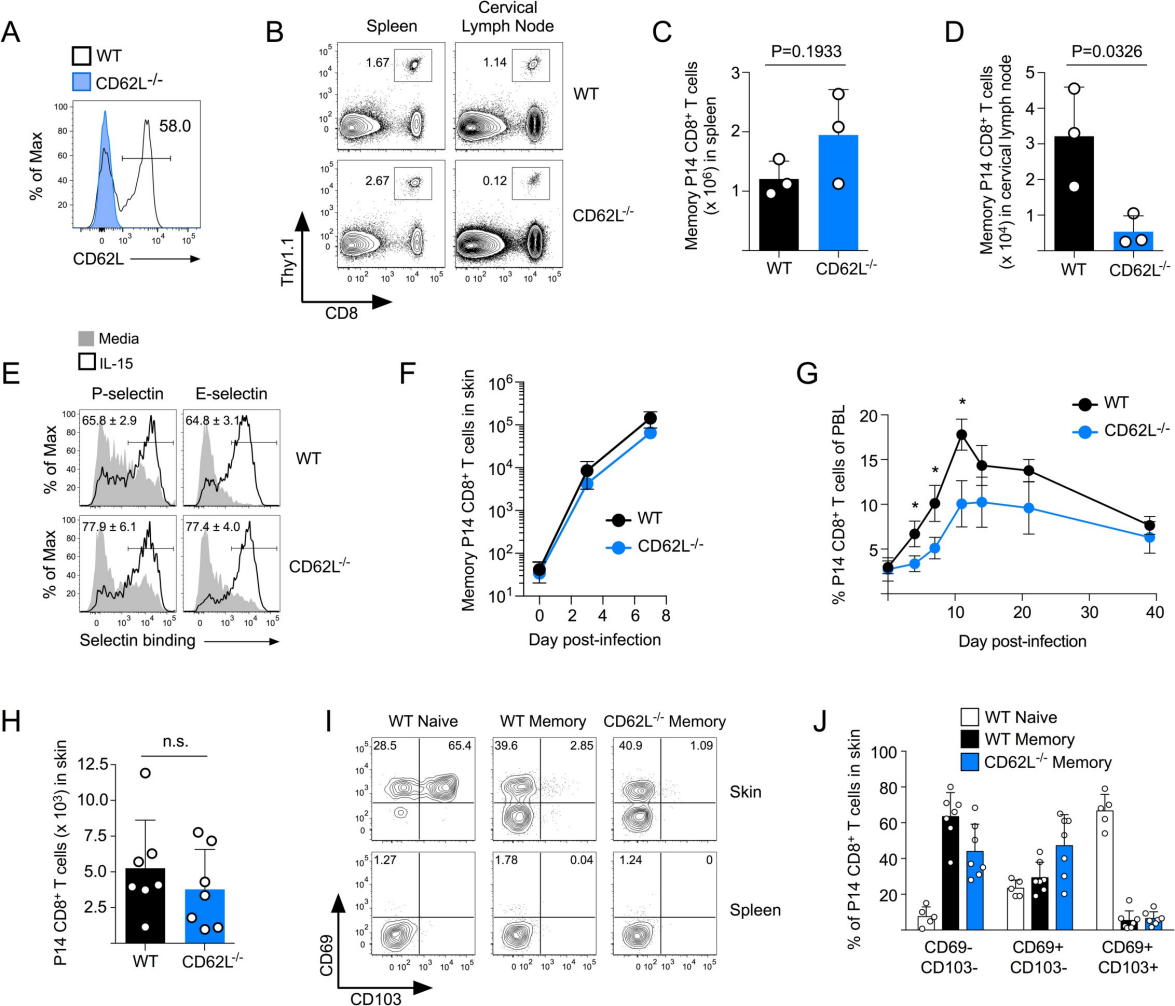
Memory CD8^+^ T cells traffic into the skin independent of CD62L, then express CD69 following VacV infection. (A) Expression of CD62L on WT and CD62L^-/-^ memory P14 CD8^+^ T cells on day 60 post-LCMV infection. (B) WT and CD62L^-/-^ memory P14 CD8^+^ T cells were identified in the spleen and cervical lymph node by flow cytometry. (C,D) Quantification of total numbers of memory P14 CD8^+^ T cells shown in (B). (E) WT or CD62L^-/-^ memory P14 CD8^+^ T cells from (A) were stimulated with IL-15 and the capacity to bind to P- and E-selectin was quantified. (F) LCMV-immune mice containing WT or CD62L^-/-^ memory P14 CD8^+^ T cells were infected with VacV-GP33 on the left ear skin and trafficking of the memory cells into the skin was quantified on days 3 and 7 post-infection. (G) Same as (F) except the expansion of the memory P14 CD8^+^ T cells was analyzed in the peripheral blood. *P<0.01 (H) Quantification of total numbers of WT and CD62L^-/-^ memory P14 CD8^+^ T cells in the skin on day 40 post VacV-GP33 skin infection. (I) Expression of CD69 and CD103 on naïve or memory P14 CD8^+^ T cells in the skin on day 40 post VacV-GP33 skin infection. (J) Quantification of (I).

Because we found that naïve CD62L^-/-^ CD8^+^ T cells were activated following systemic LCMV infection, we next evaluated whether they became functional circulating memory cells. At 50 days post LCMV infection, CD62L was expressed on ~50% of circulating WT memory CD8^+^ T cells (**Fig 3A**). Both WT and CD62L^-/-^ memory P14 CD8^+^ T cells were largely CD127+/CD27+/KLRG1- and produced IFNγ following stimulation with GP_33-41_ peptide. (**S2A–S2F Fig**). As predicted [27], there were fewer CD62L^-/-^ memory CD8^+^ T cells in lymph nodes compared to WT controls, but similar numbers were found in the spleen (**Fig 3B–3D**). Because CD62L^-/-^ memory CD8^+^ T cells that formed after LCMV infection were functional, we next tested whether CD62L-mediated lymph node surveillance was also required for memory CD8^+^ T cells to respond to viral skin infection. Both WT and CD62L^-/-^ memory CD8^+^ T cells synthesized ligands for P- and E-selectin following stimulation with IL-15 (**Fig 3E**), which we have previously demonstrated to be essential for memory CD8^+^ T cell trafficking into the skin following viral infection [14]. Indeed, CD62L^-/-^ memory CD8^+^ T cells rapidly trafficked into the skin following VacV-GP33 infection similar to WT cells (**Fig 3F**), even though secondary expansion of CD62L^-/-^ memory CD8^+^ T cells in the circulation was significantly diminished (**Fig 3G**). Nevertheless, similar numbers of both WT and CD62L^-/-^ memory P14 CD8^+^ T cells remained in the skin on day 40 following the resolution of VacV infection (**Fig 3H**). These data demonstrate that lymph node surveillance mediated by CD62L is required for naïve CD8^+^ T cells to become activated following a primary VacV infection of the skin, but not for memory CD8^+^ T cells to rapidly infiltrate and accumulate in the skin during a secondary viral infection.

We and others have shown that following activation, naïve antigen-specific CD8^+^ T cells differentiate into largely CD69+/CD103+ T_RM_ after the resolution of VacV skin infection. In contrast, most memory CD8^+^ T cells isolated from the skin at day 40 post VacV infection did not express CD103 and only a subset expressed CD69 (**Fig 3I and 3J**). CD69+ memory P14 CD8^+^ T cells in the skin also expressed higher levels of core 2 O-glycans (required for synthesis of P- and E-selectin ligands [28]) and CD44, whereas both the CD69+ and CD69- subsets expressed low levels of CD122 compared to memory CD8^+^ T cells in the spleen (**S3A–S3E Fig**). Notably, CD62L^-/-^ memory CD8^+^ T cells displayed a similar phenotype and both WT and CD62L^-/-^ memory P14 CD8^+^ T cells isolated from the skin produced IFNγ and degranulated when stimulated with GP_33-41_ peptide (**S3F–S3H Fig**). Collectively, these data suggest that circulating memory CD8^+^ T cells traffic directly into the skin in a CD62L-independent manner to provide protective immunity against viral infection, but are limited in their capacity to differentiate into canonical CD103+/CD69+ T_RM_ CD8^+^ T cells.

### CD69+ memory CD8^+^ T cells that form in the skin following secondary challenge are tissue-resident

Intravenous labeling with fluorescent antibody is often used to distinguish between immune cells in the circulation and those residing in non-lymphoid tissues such as the skin [29]. To first evaluate whether the memory CD8^+^ T cells in the skin were re-circulating or could potentially be tissue-resident, we again infected LCMV-immune mice with VacV-GP33 and on day 40 post-infection, delivered anti-CD8β antibody by intravenous injection for 3 minutes prior to isolating memory P14 CD8^+^ T cells from the skin and blood (**S4A Fig**). Memory P14 CD8^+^ T cells in the circulation were uniformly labeled with the CD8β antibody, whereas most of the memory P14 CD8^+^ T cells in the skin did not become labeled (**S4B Fig**). However, further analysis revealed that ~30–50% of the CD69- memory CD8^+^ T cells the skin were labeled with the antibody, but most of the CD69+ subset was protected (**S4B and S4C Fig**). These data suggest that the CD69- memory CD8^+^ T cells isolated from the skin were likely a re-circulating population, whereas the CD69+ subset was confined to the skin microenvironment.

**Fig 4:**
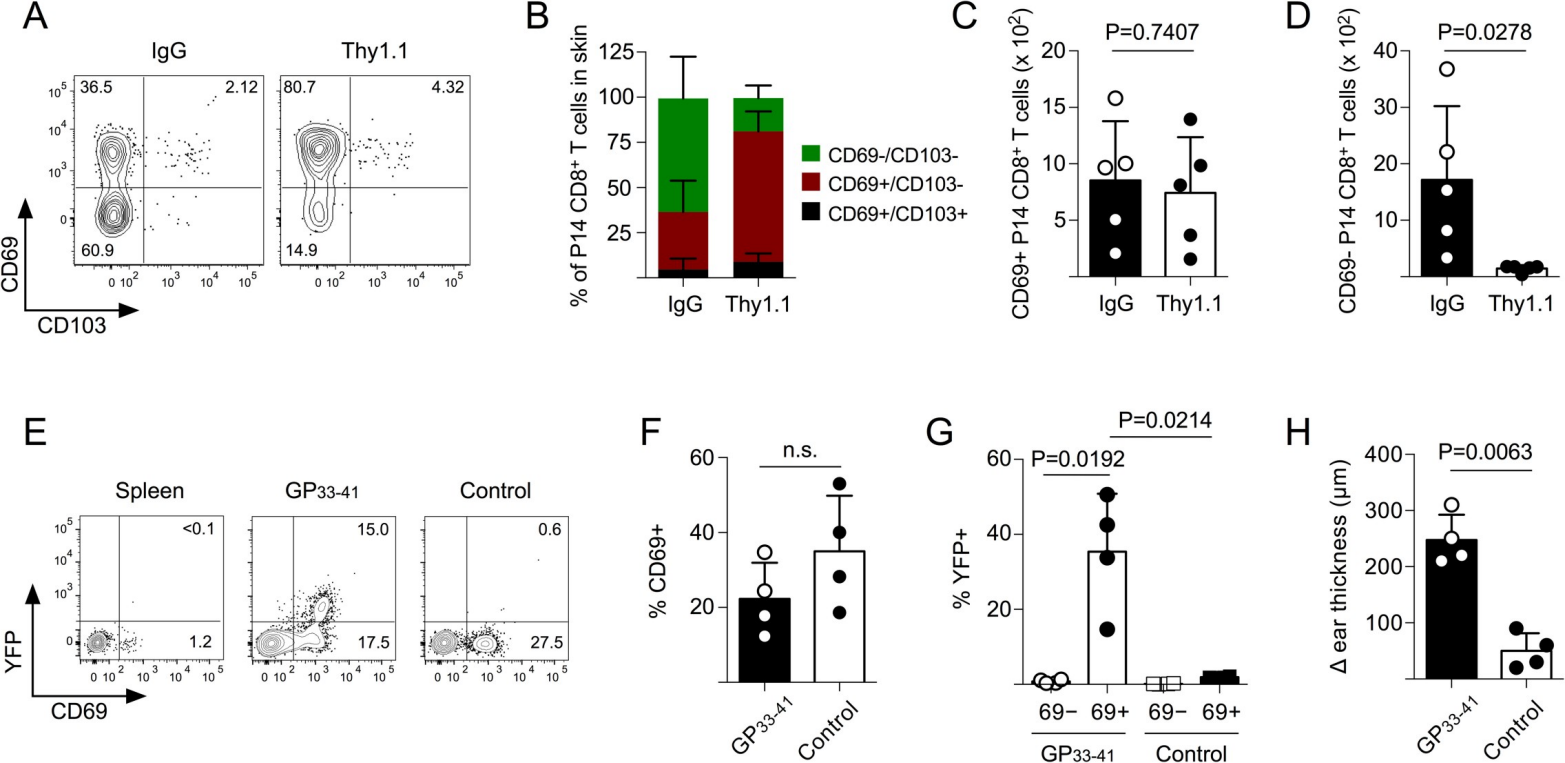
CD69+ memory CD8^+^ T cells that remain in the skin are protected from antibody-mediated depletion and produce IFNγ following topical peptide challenge. (A) LCMV immune mice were infected with VacV-GP33. On day 40 post VacV-GP33 infection, mice received control IgG or anti Thy1.1 antibody as described in *Materials and Methods*. Seven days later, memory Thy1.1+ P14 CD8^+^ T cells from the skin were analyzed for expression of CD69 and CD103. (B) Quantification of frequencies of skin P14 CD8^+^ T cells from (A). (C,D) Quantification of total numbers of (C) CD69+ and (D) CD69- memory P14 CD8^+^ T cells from (A). (E) Naïve YFP-IFNγ P14 CD8^+^ T cells were transferred into B6 mice and infected with LCMV. On day 60 post-infection, mice were then infected with VacV-GP33 on the left ear skin. On day 40 after the VacV-GP33 infection, the ear skin was challenged with either GP_33-41_ peptide or control peptide (NP_396-404_). Expression of IFNγ (YFP) was analyzed 6 hours post-peptide challenge. (F,G) Quantification of (F) CD69 expression and (G) YFP expression from (E). (H) Same mice as (E) except ear skin swelling was quantified at 6 hours post-peptide challenge.

To further confirm that the CD69+ memory CD8^+^ T cells were resident, we next utilized a Thy1.1 depleting antibody, which eliminates Thy1.1 expressing cells from the circulation, but not T_RM_ in the skin [18]. Following administration, secondary memory CD8^+^ T cells were readily depleted from the circulation, but were largely, although not completely, protected in the skin (**S5A–S5E Fig**). Similar to the result acquired by IV labeling, the memory P14 CD8^+^ T cells in the skin that were eliminated by the Thy1.1 depleting antibody were mostly the CD69-/CD103- subset (**Fig 4A–4D**), whereas cells that expressed CD69 (both CD103+/-) were protected, suggesting that memory CD8^+^ T cells that expressed CD69 in the skin following clearance of a secondary viral infection were resident in the non-lymphoid tissue.

Following TCR stimulation, antigen-specific T_RM_ CD8^+^ T cells that form following a primary infection rapidly express IFNγ and mediate local inflammation in the tissue microenvironment [12, 13, 18]. To determine whether the CD69+ cells in the skin that formed from circulating memory CD8^+^ T cells would also cause antigen-specific inflammation and/or produce IFNγ in vivo, we generated YFP-IFNγ reporter P14 CD8^+^ T cells [30]. Mice harboring T_RM_ that formed from circulating memory YFP-IFNγ P14 CD8^+^ T cells were challenged with GP_33-41_ peptide or control peptide and expression of YFP was analyzed 6 hours later. Topical challenge with antigenic peptide, but not control peptide, caused CD69+ P14 CD8^+^ T cells in the skin to produce IFNγ (**Fig 4E**). Although the frequency of CD69+ memory CD8^+^ T cells in the skin did not change following peptide challenge, IFNγ expression was found exclusively in the CD69+ subset (**Fig 4F and 4G**). Finally, GP_33-41_ peptide challenge also caused tissue swelling (**Fig 4H**), suggesting that the T_RM_ derived from circulating memory CD8^+^ T cells were positioned to rapidly respond to antigens in the periphery and initiate local inflammatory responses. Collectively, these data suggest that the CD69+ P14 CD8^+^ T cells that formed from circulating memory CD8^+^ T cells exhibit functional features of bona fide T_RM_ CD8^+^ T cells.

### Circulating memory CD8^+^ T cells differentiate into CD69+ T_RM_ in an antigen-specific manner

**Fig 5:**
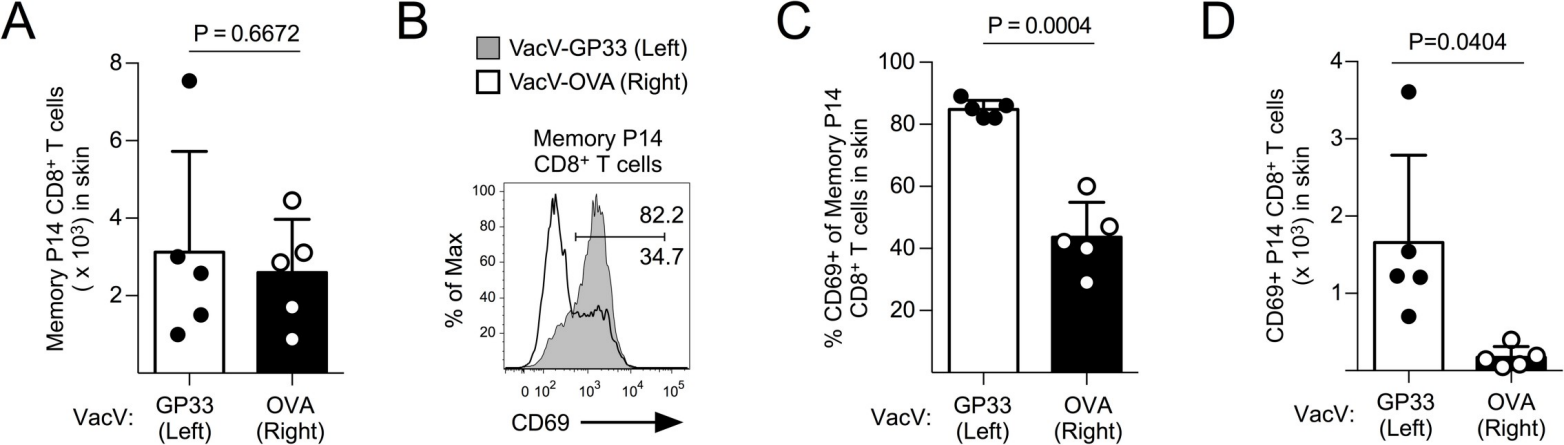
Circulating memory CD8^+^ T cells become CD69+ T_RM_ following local antigen recognition in the skin. (A) LCMV-immune mice were infected with VacV-GP33 on the left ear skin and VacV-OVA on the right. On day 15 post-infection, total number of memory P14 CD8^+^ T cells in the skin were quantified. (B) CD69 expression on memory P14 CD8^+^ T cells from (A). (C) Quantification of (B). (D) On day 40 post-infection, the number of CD69+ memory P14 CD8^+^ T cells was quantified in the left and right ear skin.

Previously, we have demonstrated that local antigen recognition in the skin significantly increases the generation of antigen-specific T_RM_ CD8^+^ T cells following a primary VacV infection [18]. To determine whether memory CD8^+^ T cells that traffic directly into VacV-infected skin also relied on local antigen recognition to become a CD69+ T_RM_, we co-infected LCMV-immune mice with VacV-GP33 on the left ear skin and VacV expressing the model antigen OVA_257-264_ (VacV-OVA) on the right, as these viral infections are restricted to the skin microenvironment and do not become systemic [18]. Notably, using this strategy, we have also previously shown that GP33-specfic memory CD8^+^ T cells in LCMV-immune animals provide protective immunity in the VacV-GP33 infected skin, but not the VacV-OVA infected skin [15]. On day15 post-infection, memory P14 CD8^+^ T cells had trafficked into the skin of both viral infections (**Fig 5A**), but expressed more CD69 when cognate antigen was present in the VacV-infected skin microenvironment (**Fig 5B and 5C**). Furthermore, on day 40 post-infection, more CD69+ memory P14 CD8^+^ T cells were present in the skin infected with VacV-GP33 compared to the skin infected with VacV-OVA (**Fig 5D**), demonstrating that local antigen recognition in the skin increased the retention of antigen-specific secondary T_RM_ CD8^+^ T cells. Collectively, these data show that like naïve CD8^+^ T cells, circulating memory CD8^+^ T cells become CD69+ T_RM_ primarily at the site of viral infection in an antigen-specific manner.

### CD69+ T_RM_ cells that form following secondary viral skin infection are derived from reactivated T_CM_ CD8^+^ T cells

**Fig 6:**
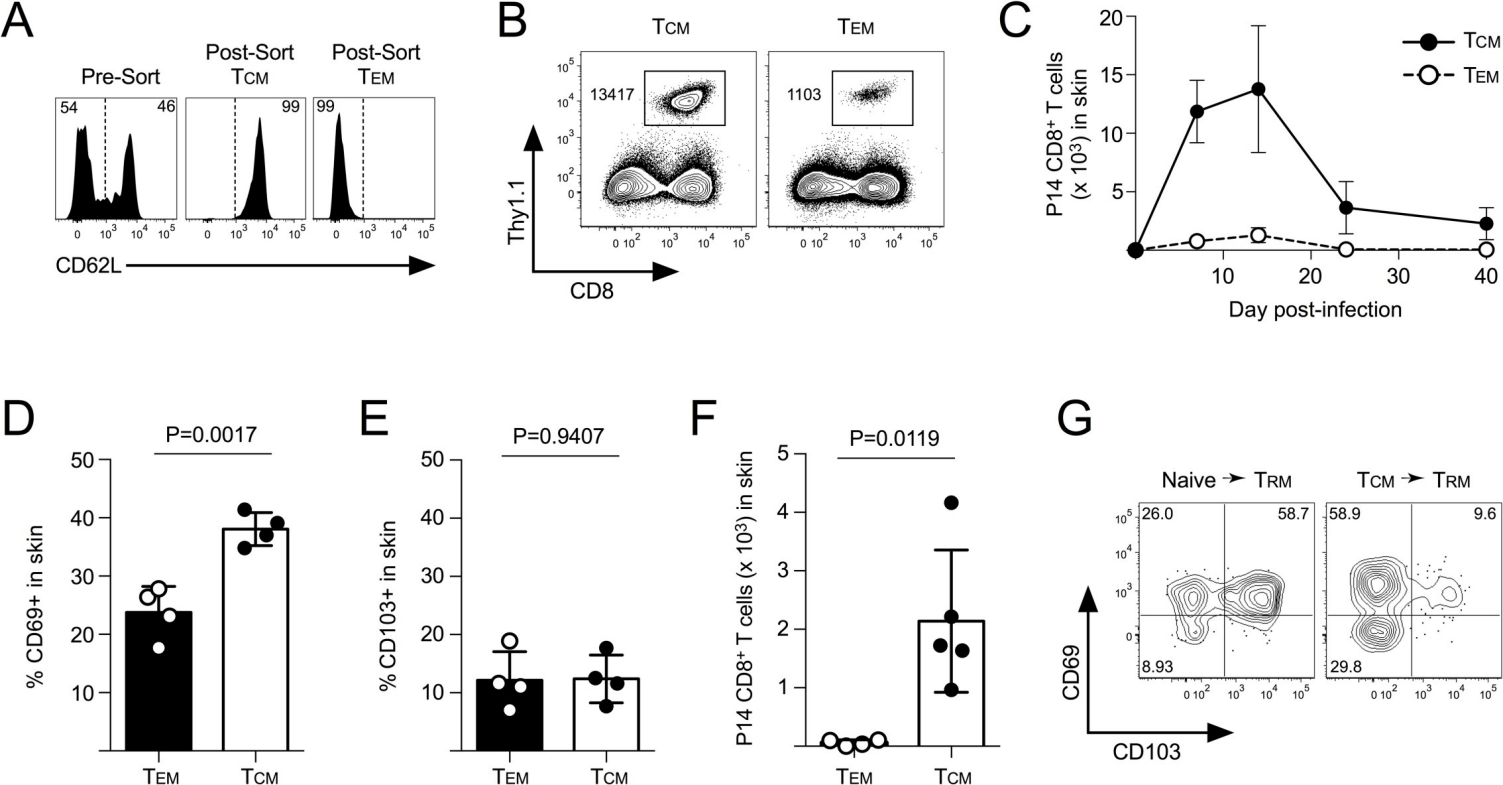
T_CM_ CD8^+^ T cells become CD69+ T_RM_ following VacV infection. (A) 1 x 10^5^ T_CM_ (CD62L+) or T_EM_ (CD62L-) memory P14 CD8^+^ T cells were sorted by FACS and transferred into B6 mice, which were then infected on the left ear skin with VacV-GP33. (B) On day 7 post-infection, trafficking of memory CD8^+^ T cells into the skin was analyzed. (C) The total number of memory P14 CD8^+^ T cells in the skin from (B) was quantified over time. (D, E) Expression of (D) CD69 and (E) CD103 was analyzed in the skin on day 14 post-infection. (F) Total number of memory P14 CD8^+^ T cells in the skin on day 40 post-infection. (G) Expression of CD69 and CD103 on P14 CD8^+^ T cells in the skin on day 40 post VacV-GP33 skin infection that originated from either naïve or T_CM_ populations.

Memory CD8^+^ T cells in the circulation can be broadly defined as being either T_EM_ or T_CM_ by the expression of receptors required for lymph node trafficking across high endothelial venules [31, 32]. To determine which of these subsets differentiated into CD69+ T_RM_ that formed in the skin, we sorted T_CM_ and T_EM_ P14 CD8^+^ T cells based on expression of CD62L and transferred equal numbers into naïve mice followed by infection with VacV-GP33 (**Fig 6A**). T_CM_ expanded and trafficked into the skin better than T_EM_ CD8^+^ T cells throughout the course of the VacV infection (**Fig 6B and 6C**). Reactivated T_CM_ CD8^+^ T cells expressed slightly more CD69 in the skin than T_EM_ CD8^+^ T cells, whereas both subsets expressed equally low levels of CD103 (**Fig 6D and 6E**). By day 40 post-infection, a stable population of P14 CD8^+^ T cells that originated from T_CM_ formed in the skin, whereas the transferred T_EM_ CD8^+^ T cells were essentially undetectable (**Fig 6F**). In contrast to T_RM_ derived from naïve CD8^+^ T cells, T_CM_-derived T_RM_ CD8^+^ T cells were also largely CD69+/CD103- (**Fig 6G**). Overall, these data, along with our previous study [15] demonstrate that circulating T_CM_ are both the major tissue-trafficking memory CD8^+^ T cells subset and become CD69+ T_RM_ in the skin following reactivation.

### Naïve CD8^+^ T cells express CD103 and become tissue-residents better than circulating T_CM_ following viral skin infection

**Fig 7:**
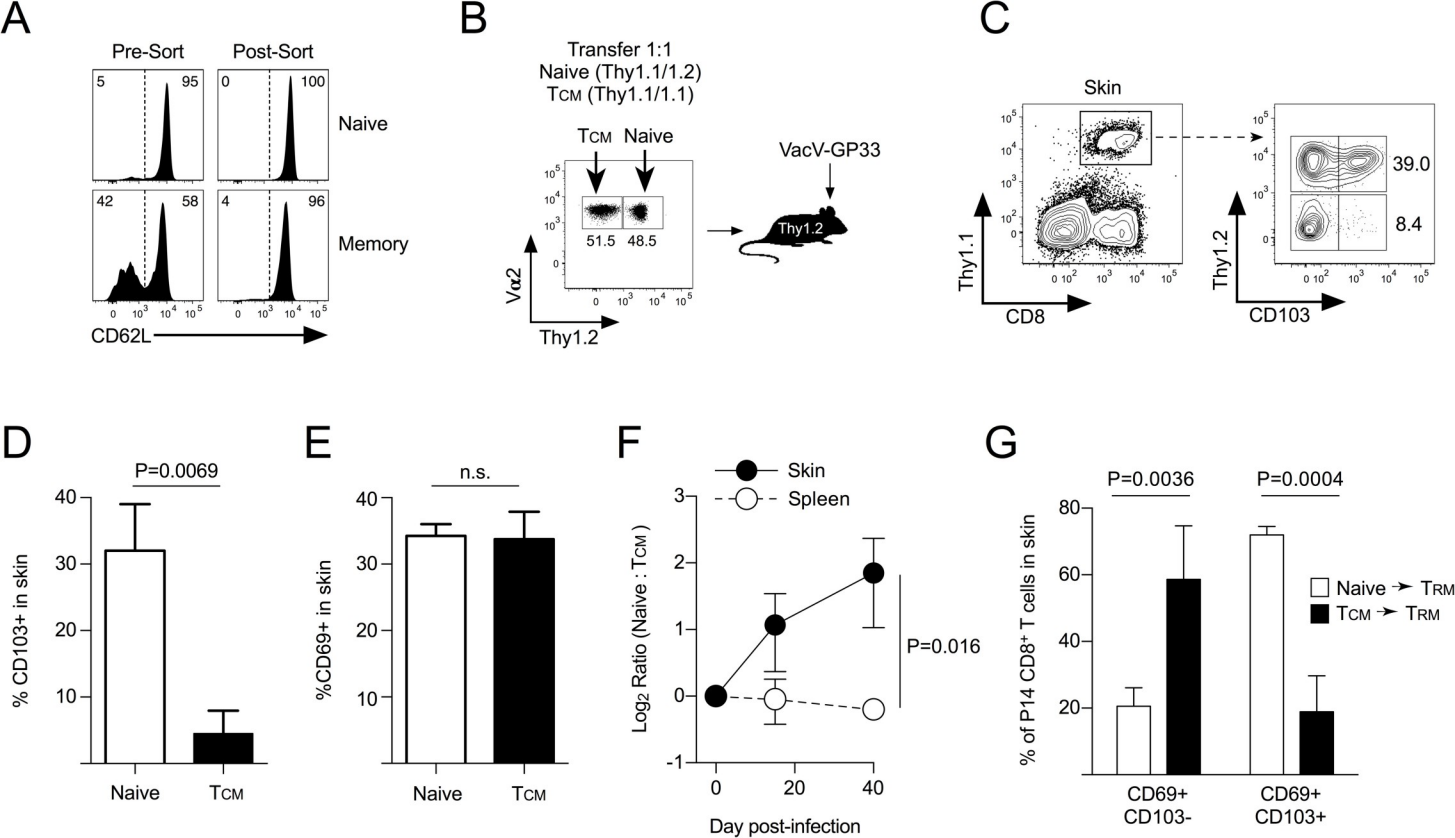
Naïve CD8^+^ T cells form tissue-residents following VacV skin infection better than circulating T_CM_ CD8^+^ T cells. (A) CD62L+ naïve or T_CM_ P14 CD8^+^ T cells were sorted by FACS. (B) Equal numbers of naïve (Thy1.1/1.2) and T_CM_ (Thy1.1/1.1) CD8^+^ T cells from (A) were transferred into naïve B6 mice (Thy1.2/1.2) and infected with VacV-GP33 on the left ear skin. (C) Expression of CD103 on day 15 post-infection. (D) Quantification of (C). (E) Expression of CD69 on day 15 post-infection. (F) Log_2_ ratio of naïve to T_CM_ P14 CD8^+^ T cells in the spleen and VacV infected skin. (G) On day 40 post-infection, expression of CD69 and CD103 was quantified on P14 CD8^+^ T cells in the skin that originated from either naïve or T_CM_ CD8^+^ T cells.

Because the previous experiment demonstrated that T_CM_ became the CD69+ T_RM_ that formed in the skin during secondary viral infection, we next tested whether T_CM_ or naïve CD8^+^ T cells exhibited a greater capacity to express CD103 and/or differentiate into T_RM_ following VacV infection. Equal numbers (5 x 10^4^) of purified naïve (Thy1.1/1.2) and T_CM_ (Thy1.1/1.1) P14 CD8^+^ T cells were transferred into naïve (Thy1.2/1/2) B6 mice and infected on the left ear skin with VacV-GP33 (**Fig 7A and 7B**). Both CD8^+^ T cell populations trafficked into the skin on day 15 post-infection and previously naïve CD8^+^ T cells began expressing CD103 and CD69 [18], but T_CM_ CD8^+^ T cells in the same skin microenvironment expressed only CD69 (**Fig 7C–7E**). In addition, previously naïve P14 CD8^+^ T cells were found at increased frequencies compared to T_CM_ in the infected skin microenvironment on days 15 and 40 post VacV infection (**Fig 7F**), but both populations were equally represented in the spleen, demonstrating that the competitive advantage was occurring specifically in the VacV-infected skin microenvironment. Finally, on day 40 post-infection, the naïve CD8^+^ T cells formed mature CD69+/CD103+ T_RM_ CD8^+^ T cells, but the T_CM_ remained largely CD69+/CD103- (**Fig 7G**). Overall, these data reveal that on a per cell basis, naïve CD8^+^ T cells express more CD103 after infiltrating the VacV infected skin and more efficiently become mature T_RM_ compared to T_CM_ CD8^+^ T cells.

**Fig 8:**
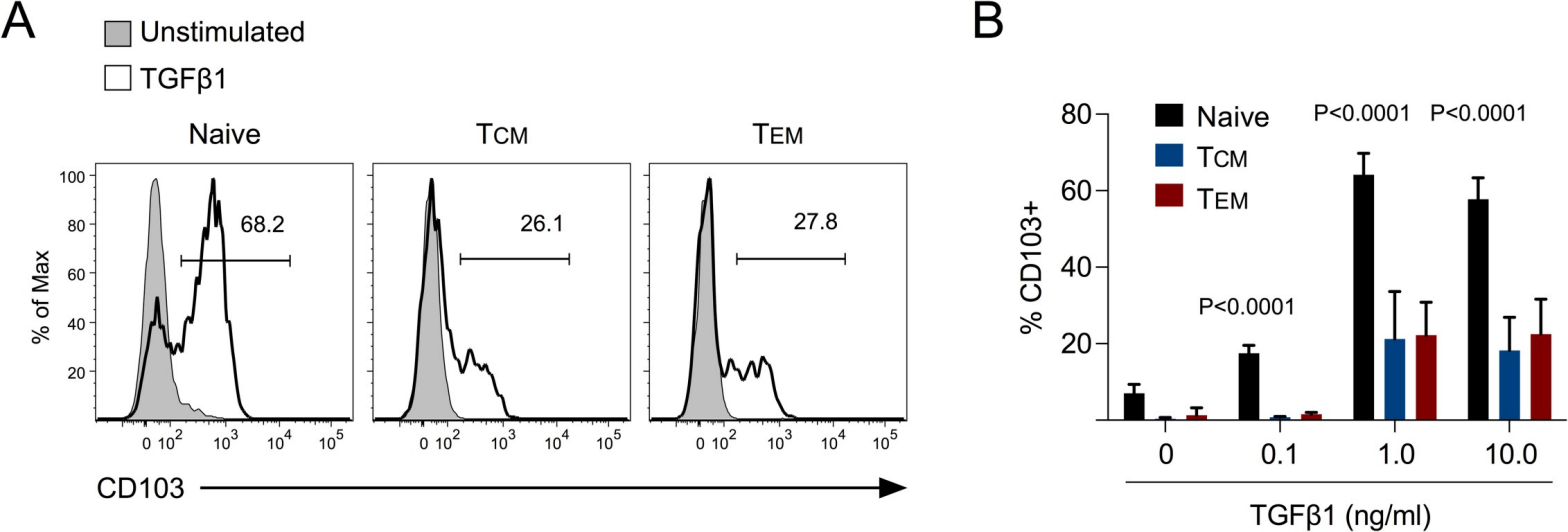
Previously naïve CD8^+^ T cells express more CD103 than memory CD8^+^ T cells when stimulated with TGF-β following VacV skin infection. (A) Naïve (Thy1.1/1.2) and either purified T_CM_ or T_EM_ (Thy1.1/1.1) P14 CD8^+^ T cells were co-transferred into naïve B6 mice that were then infected on the left ear skin with VacV-GP33. On day 10 post-infection, splenocytes were stimulated with 1 ng/ml TGF-β1 for 48 hours and expression of CD103 was analyzed. (B) Same experimental design as (A) except CD103 expression was quantified following stimulation with the indicated concentration of TGF-β1.

TGF-β is a known stimulator of CD103 expression and both TGFβRII^-/-^ and CD103^-/-^ CD8^+^ T cells exhibit reduced T_RM_ differentiation during viral infection of the skin [20]. Because most T_CM_ did not express CD103 in the skin following VacV skin infection, we next assessed whether this was due to limited capacity to express this integrin downstream of TGF-β receptor signaling. On day 10 post-infection, previously naïve, T_CM_, and T_EM_ P14 CD8^+^ T cells from the spleen were stimulated ex vivo with increasing concentrations of TGF-β1. In agreement with our data from Fig 7C and 7D, ~60% of previously naïve CD8^+^ T cells expressed CD103 when stimulated with TGF-β (**Fig 8A and 8B**). In contrast, only ~25% of T_CM_ and T_EM_ CD8^+^ T cells expressed CD103 following TGF-β stimulation. This result was not specific to VacV skin infection, as both T_CM_ and T_EM_ CD8^+^ T cells reactivated by systemic LCMV infection also exhibited reduced TGF-β-stimulated CD103 expression compared to previously naïve CD8^+^ T cells (**S6A and S6B Fig)**. Thus, these data suggest that TGF-β-mediated CD103 expression is largely a feature of previously naïve CD8^+^ T cells that become activated following infection.

### Circulating memory CD8^+^ T cells use cytolysis to provide protective immunity against VacV skin infection

**Fig 9:**
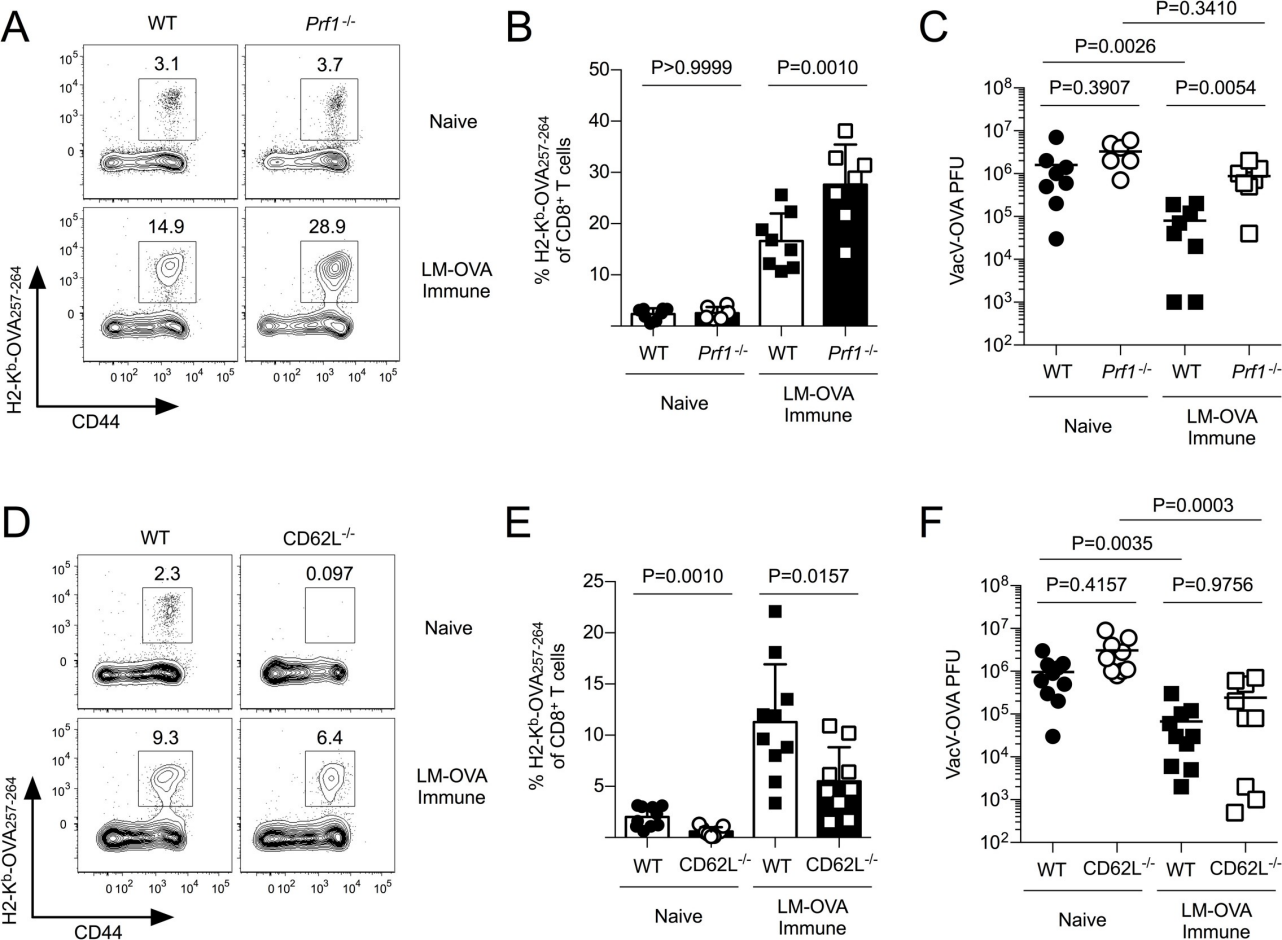
Circulating memory CD8^+^ T cells require perforin, but not CD62L, to provide protective immunity against VacV skin infection. (A) WT or *Prf1*^-/-^ B6 mice were infected with 2 x 10^7^ CFU of attenuated LM-OVA. On day 40 post-infection, LM-OVA-immune animals or naïve controls were infected with 1 x 10^6^ PFU VacV-OVA on the left ear skin and the frequency of H2-K^b^ OVA_257-264_ specific CD8^+^ T cells were quantified in the blood. (B) Quantification of (A). (C) Viral titers in the ear skin of mice from (A) on day 7 post-infection. (D) WT and *Lsel*^-/-^ mice were infected with attenuated LM-OVA (2 x 10^7^ CFU). On day 40 post-infection, naïve controls or LM-OVA immune mice were infected with VacV-OVA on the left ear skin. On day 7 post-VacV-OVA skin infection, H2-K^b^-OVA_257-264_ specific CD8^+^ T cells were analyzed in the blood. (E) Quantification of (D). (F) Viral load was quantified from the ear skin.

It has been reported previously that memory CD8^+^ T cells express granzyme B following exposure to inflammatory cytokines and/or after entering non-lymphoid tissues such as the lung [33, 34]. Following VacV-GP33 skin infection, memory P14 CD8^+^ T cells that initially trafficked into the skin expressed granzyme B, as did memory cells undergoing proliferation in the draining lymph node. (**S7A Fig**). Because memory CD8^+^ T cells that trafficked into VacV-infected skin expressed high levels of granzyme B, we next tested whether memory CD8^+^ T cells required cytolytic function to provide protective immunity against the viral infection. We vaccinated WT and perforin (*Prf1)*^-/-^ mice with attenuated (*ActA*^-/-^) *Listeria monocytogenes* expressing OVA (LM-OVA), rather than LCMV, as *Prf1*^-/-^ mice are unable to clear an acute LCMV infection [35]. Following VacV-OVA skin infection, H2-K^b^-OVA_257-264_ specific memory CD8^+^ T cells expanded slightly more in LM-OVA immune *Prf1*^-/-^ mice compared to WT controls (**Fig 9A and 9B**), but provided less protection against the virus (**Fig 9C**). In agreement with Fig 3, CD62L^-/-^ OVA-specific CD8^+^ T cells in LM-OVA immune mice expanded less than WT controls following VacV-OVA challenge (**Fig 9D and 9E**), but still provided protective immunity against viral skin infection (**Fig 9F**). This result was not specific to OVA-specific memory CD8^+^ T cells or vaccination with attenuated *Listeria*, as CD62L^-/-^ GP33-specific circulating memory CD8^+^ T cells that formed following LCMV infection also provided protective immunity against VacV-GP33 infection of the skin similar to WT controls (**S7B–S7E Fig**). Collectively, these data demonstrate that circulating memory CD8^+^ T cells require perforin, but not CD62L-mediated lymph node surveillance to provide protective immunity in the skin.

## Discussion

T_RM_ CD8^+^ T cells are believed to play an important role in host defense against pathogens, but have also been suggested to be the dysfunctional culprit in a variety of inflammatory diseases of the skin and perhaps other non-lymphoid tissues [36, 37]. Thus, an understanding of both the cell intrinsic mechanisms, as well as the cell extrinsic microenvironment inflammatory factors that ultimately control the formation of these cell populations is highly relevant for a variety of diseases and pathological conditions. Most studies to date have focused on the capacity for T_RM_ CD8^+^ T cells to develop from naïve CD8^+^ T cells following a primary infection in mice, where no protective adaptive immunity against the pathogen is present. Notably, there are essentially no T_RM_ CD8^+^ T cells in laboratory mice prior to any type of experimental manipulation, whereas human skin is rich in a heterogeneous mixture of CD8^+^ and CD4^+^ T cells exhibiting a variety of phenotypes [22]. This observation suggests that exposure to environmental antigens and pathogens throughout life is continually shaping the composition of the skin T cell repertoire [38]. Because most of our current knowledge of the mechanisms controlling T_RM_ formation have been studied in the context of a primary infection, understanding how pre-existing immunological memory and dynamic host-pathogen interactions within the local tissue microenvironment impact T_RM_ differentiation has remained largely unknown.

Recent studies by us and others suggest that antigen recognition in non-lymphoid tissues such as the skin and lung will significantly increase the formation of T_RM_ following a primary infection [18, 39–42]. Indeed, we also found that memory CD8^+^ T cells became enriched in the skin microenvironment where antigen recognition occurred during a secondary challenge. Interestingly, however, we also found that most circulating memory CD8^+^ T cells that infiltrate the skin during VacV infection express CD69 following antigen recognition, but are unable to express CD103 when stimulated with TGF-β. Although the generation of a local inflammatory environment is clearly essential for T cell recruitment prior to T_RM_ differentiation, it has also been demonstrated that antigen recognition and some pro-inflammatory cytokines such as IL-12 and IFNβ will inhibit CD103 expression [23, 43]. In contrast to our findings, it has recently been reported that CD69+/CD103+ T_RM_ CD8^+^ T cells are generated from circulating memory CD8^+^ T cells following HSV-1 skin infection [44]. Interestingly, we and others have shown that local antigen recognition in the skin is critical for T_RM_ CD8^+^ T cells to form following poxvirus infection [18, 39, 45], whereas antigen-specific T_RM_ formation does not seem to occur during skin infection with HSV-1 [9]. Because here we also show that CD69+ T_RM_ are generated by circulating memory T cells in an antigen-specific manner, this suggests that antigen recognition in the skin is critical for the formation of this population following poxvirus infection, but may not form and/or be retained during HSV-1 skin infection. Thus, it seems that both antigen recognition and the cytokine milieu within the tissue microenvironment act in concert to ultimately control the extent of T_RM_ differentiation, but how these diverse signaling pathways are integrated in vivo is still largely undefined. Nevertheless, our data support a model where expression of CD69 identifies skin T_RM_ that received a local antigen recognition event, but not a TGF-β (or perhaps additional cytokines) signal to transition into a CD103+/CD69+ T_RM_.

Our study also highlights that the capacity for memory CD8^+^ T cells to home into and survey peripheral lymph nodes does not play a major role during a secondary recall response against a local skin infection and that the primary feature of T_CM_ that allows this memory subset to provide protective immunity is their capacity to rapidly and specifically traffic into inflamed non-lymphoid tissues. Another recent report demonstrated that KLRG1+, CX3CR1+ T_EM_ CD8^+^ T cells are essentially confined to the circulation and are unable to infiltrate non-lymphoid tissues during steady-state homeostatic trafficking [46]. In addition, it has also been recently reported that T_CM_ CD8^+^ and CD4^+^ T cells from humans express significant levels of non-lymphoid tissue-homing addressins and induce skin inflammation in a mouse model of dermatitis [47]. These studies agree with our data demonstrating that T_CM_ are the major tissue trafficking subset that provides protection against VacV skin infection [15] and also are precursors to CD69+ T_RM_. In contrast, highly cytolytic T_EM_ CD8^+^ T cells provide more protective immunity than T_CM_ against systemic infections that reach the circulation and/or spleen (i.e., when pathogens are delivered to mice intravenously or into the peritoneal cavity) [48, 49]. Interestingly, we found that circulating memory CD8^+^ T cells also require cytolysis to control VacV infection of the skin and limit inflammation, which is in contrast to T_RM_ CD8^+^ T cells, which have been proposed to provide their protective benefit through local production of IFNγ [12, 13]. It will be of interest to determine whether circulating memory CD8^+^ T cells use cytolysis as the primary mechanism to control other intracellular pathogens in non-lymphoid tissues or if the production of cytokines (e.g. IFNγ, TNFα) is required to protect against some types of infections. Altogether, our findings suggest that the circulating T_CM_ CD8^+^ T cells function as the first responders against infections in peripheral tissues, ultimately controlling and eliminating viral infection.

In summary, our results demonstrate that T_CM_ CD8^+^ T cells traffic into non-lymphoid tissue microenvironments following viral infection in a CD62L-independent fashion and protect against poxvirus using perforin-mediated cytotoxicity. Our finding that the majority of the memory CD8^+^ T cells initially recruited into the site of viral infection (by day 3) had not proliferated strongly suggests that the primary feature of T_CM_ that allows this memory subset to provide rapid protective immunity is their ability to infiltrate inflamed non-lymphoid tissues, rather than the re-activation/proliferative expansion in draining lymph nodes. In fact, recent experimental evidence suggests that terminally differentiated T_EM_ CD8^+^ T cells are largely confined to the circulation and not actively surveying non-lymphoid tissues [15, 46, 50] and thus, the long-standing model describing the trafficking potentials of T_CM_ and T_EM_ CD8^+^ T cells subsets continues to be refined. Overall, our findings provide new insight regarding the protective mechanisms used by circulating memory CD8^+^ T cells during a secondary viral infection in peripheral tissues, but also reveals important information about how “prime-boost” vaccination strategies of generating circulating memory CD8^+^ T cells followed by a local heterologous challenge will generate antigen-specific secondary memory T cell populations in non-lymphoid tissues such as the skin.

## Materials and methods

### Ethics statement

All animal experiments were conducted in accordance with the Animal Welfare Act and the recommendations in the Guide for the Care and Use of Laboratory Animals of the National Institutes of Health. Approved by the OHSU Institutional Animal Care and Use Committee (Protocol Number IP00715) and Institutional Biosafety Committee (Registration Number IBC-13-33).

### Mice and pathogens

C57BL/6N mice were purchased from NCI/Charles River. Perforin^-/-^ [51] and IFN-γ YFP reporter [30] mice were purchased from Jackson Laboratories. P14 TCR-transgenic [52] and *Lsel*^-/-^ [26] mice have been previously described and were maintained by sibling x sibling mating. For adoptive transfers, 1.0–5.0 x 10^4^ Thy1.1+ P14 CD8^+^ T cells from either blood or spleen were injected intravenously in 200 μl of PBS. LCMV-Armstrong was delivered i.p. (2 x 10^5^ PFU) in 200 μl of PBS. Attenuated (ActA^-/-^) *Listeria monocytogenes* expressing ovalbumin (LM-OVA) was delivered intravenously (1 x 10^7^ CFU) in 200 μl of PBS. Vaccinia virus (VacV) expressing GP_33-41_ (VacV-GP33) and ovalbumin_257-264_ (VacV-OVA) have been previously described and were propagated using BSC-40 cells and standard protocols [53, 54]. Infections with VacV were performed on anesthetized mice by placing 1–5 x 10^6^ PFU of virus in 10 μl of PBS on the ventral side of the ear pinna and then poking the virus coated skin 25 times with a 27G needle. For depletion of Thy1.1 expressing CD8^+^ T cells from the circulation, mice were treated with 1 μg of control rat IgG (Sigma) or anti-Thy1.1 antibody (clone 19E12, BioXCell) 1–3 times in 200 μl of PBS by i.p. injection.

### Quantification of VacV from infected skin

Quantification of viral load in the infected skin was determined using standard plaque assays on BSC-40 cells. Briefly, infected ears were removed and homogenized in 1 ml of RPMI supplemented with 1% fetal bovine serum. Skin homogenates were then subjected to three rounds of freeze-thaw before serial dilutions were inoculated on BSC-40 cells in a 12-well plate that were then covered with 1% Seakem agarose in Modified Eagle Medium (Gibco). Plaques were visualized three days later following overnight incubation with Neutral Red dye.

### Leukocyte isolation from skin

Ears from infected mice were removed and the dorsal and ventral sides of the ear pinna were separated and allowed to incubate for 1–2 hours at 37°C with 1–2 ml HBSS (Gibco) containing CaCl_2_ and MgCl_2_ supplemented with 125 U/ml collagenase (Invitrogen) and 60 U/ml DNase-I (Sigma-Aldrich) at 37°C. Whole tissue suspensions were then generated by gently forcing the tissue through a wire mesh screen. Leukocytes were then purified from whole tissue suspensions by re-suspending the cells in 10 mL of 35% Percoll (GE Healthcare)/HBSS in 50 mL conical tubes followed by centrifugation (500 x g) for 10 minutes at room temp. To identify tissue-resident CD8^+^ T cells by intravenous labeling, Anti-CD8β antibody (clone YTS156.7.7, 3 μg) was diluted in 200 μl of PBS and delivered to mice via intravenous injection. After 3 minutes, mice were immediately sacrificed and cells from the skin were isolated as described above.

### Flow cytometry and cell sorting

H2-K^b^-OVA_257-264_ and H2-D^b^-GP_33-41_ tetramers were kindly provided by Dr. John Harty, University of Iowa. Staining for surface antigens was performed in PBS/1% fetal bovine serum for 15 minutes at 4°C. For tetramer binding, cells were incubated for 45 minutes at room temp. Data was acquired using either a BD Fortessa or BD LSRII Flow Cytometer in the OHSU Flow Cytometry Core Facility. Flow cytometry data was analyzed using FlowJo software, version 9.9 or 10. To generate purified central memory, effector memory, or naïve P14 CD8^+^ T cells, single cell suspensions of splenocytes from naïve or LCMV-immune mice were stained with Thy1.1-PE, CD62L-APC, and CD8α-FITC in 1%FBS/PBS for 15 minutes at 4°C. Cells were washed and then incubated with anti-PE magnetic beads (Miltenyi) according to the manufacturers protocol in 1%FBS/PBS for 10 minutes at 4°C. Thy1.1+ P14 CD8^+^ T cells were then enriched using the Miltenyi autoMACS Pro Separator prior to sorting. Sorting of cells was performed on a BD Influx cell sorter in the OHSU Flow Cytometry Core Facility.

### Antibodies

The following antibodies for flow cytometry and corresponding isotype controls were used in this study: CD8α (53–6.7, BioLegend), CD44 (1M7, BioLegend), CD45.2 (104, Tonbo), CD62L (MEL-14, BioLegend), Thy1.1 (OX-7, BioLegend), Thy1.2 (53–2.1, BioLegend), CD69 (H1.2F3, BioLegend), CD103 (2E7, BioLegend), CD127 (A7R34, BioLegend), KLRG1 (2F1, Tonbo), BrdU (3D4, BioLegend), Vα2 TCR (B20.1, BioLegend), IFNγ (XMG1.2, Tonbo), CD122 (5H4, BioLegend), glycosylated isoform of CD43 (1B11, BioLegend), CD107a (1D4B, BioLegend), CD107b (M384, BioLegend), CD27 (LG.3A10, BioLegend), granzyme B (GB11, BioLegend).

### Synthesis of P- and E-selectin ligands

To detect synthesis of P- and E-selectin ligands, WT and CD62L^-/-^ memory P14 CD8^+^ T cells from spleens were stimulated for 3 days with 250 ng/ml of recombinant mouse IL-15 (Peprotech). Recombinant E-selectin (5 μg/ml) and P-selectin (1.5 μg/ml) human IgG Fc chimeric proteins (R & D Systems) were incubated with cells for 30 minutes in 1% FBS/DPBS containing Ca^2+^ and Mg^2+^ (Gibco) at room temperature. Binding of selectins was detected using anti-human IgG-Fc PE (eBioscience). Cells were then stained with fluorescent antibodies as described in *Flow Cytometry and Antibodies*.

### Ex vivo peptide and TGF-β stimulation

Spleens were collected on the indicated days post-VacV infection and single cell suspensions were generated by gently forcing the tissue through a mesh screen. For detection of IFNγ, splenocytes were incubated for 5 hours with 500 nM of GP_33-41_ peptide (Bio-synthesis) in the presence of Brefeldin A (BioLegend). Intracellular cytokine stain was performed using the CytoFix/CytoPerm kit (BD Biosciences) according to the manufacturers protocol. Briefly, following the staining of surface antigens as described in *Flow Cytometry and Cell Sorting*, cells were incubated with 100 μl of Cytofix/Cytoperm for 10 minutes at 4°C and washed once with Perm/Wash buffer. Staining for IFNγ was performed in Perm/Wash buffer for 20 minutes at 4°C. For analysis of cytokine production by CD8^+^ T cells isolated from skin, single cell suspensions were incubated overnight with 1 μM of GP_33-41_ peptide and stained as described above. Degranulation of memory CD8^+^ T cells was analyzed by surface expression of CD107a/b following peptide stimulation. Single cell leukocyte suspensions isolated from skin were stimulated overnight with media containing 1 μM GP_33-41_ peptide with CD107a and CD107b antibody (0.25 μg/ml each) in the presence of monensin (BioLegend). To detect expression of CD103 on P14 CD8^+^ T cells following infection, single cell suspensions from spleens were generated as described above. Whole splenocytes were then stimulated with 0.1–10 ng/ml human TGF-β1 (Peprotech) for 48 hours.

### In vivo peptide challenge

GP_33-41_ or NP_396-404_ peptides (5 μg) were dissolved in 20 μL of 4:1 acetone/DMSO. Mice were anesthetized with isofluorane and the dissolved peptide solution was applied to both the dorsal and ventral sides of previously infected ear skin. Tissue swelling and IFNγ expression was analyzed 6 hours after challenge. Thickness of the ear pinna was measured on anesthetized mice with a dial micrometer (Ames).

### BrdU incorporation

LCMV-immune B6 mice were administered 2 mg of Brdu (Sigma) in PBS one day prior to VacV infection and provided drinking water containing 0.8 mg/ml during the 7 day course of infection. Staining of cells for BrdU incorporation was performed using the BrdU Flow kit (BD Bioscienes) according to the manufacturers protocol. Briefly, single cell suspensions of leukocytes were generated from the skin and spleen as described previously. Following staining of surface antigens, cells were treated with Cytofix/Cytoperm buffer for 30 minutes. Cells were washed with Perm/Wash Buffer and then fixed with Cytoperm Permeabilization Buffer Plus for 15 minutes. Cells were again washed with Perm/Wash buffer and were treated with Cytofix/Cytoperm buffer a second time for 10 minutes. Cells were then incubated with DNase I (0.33 mg/ml in PBS) for 75 minutes at 37°C before cells were stained with antibody against BrdU (BD Biosciences) in Perm/Wash Buffer for 30 minutes at room temperature. Cells were then washed twice with Perm/Wash Buffer and re-suspended in PBS for analysis.

### Statistical analysis

Statistical analyses were performed with Prism software (version 6.0, GraphPad Software) using either the paired or unpaired Student’s t test or ANOVA with Tukey’s post-test for significance.

## Supporting information

S1 Fig Acute LCMV-Armstrong infection generates circulating memory CD8^+^ T cells, but not T_RM_ in the skin. (A) 1 x 10^4^ naïve Thy1.1 P14 CD8^+^ T cells were transferred into naïve B6 mice which were then infected with either VacV-GP33 on the left ear skin or LCMV by i.p. injection. On day 40 post-infection, the distribution of memory P14 CD8^+^ T cells was analyzed in the indicated tissues. (B) Quantification of (A). (C) Expression of CD69 and CD103 of memory P14 CD8^+^ T cells from either the skin or spleen on day 40 post-infection. (D) Quantification of (C).
(TIF)

S2 Fig CD62L^-/-^ CD8^+^ T cells become functional circulating memory cells following acute, systemic LCMV infection. Naïve WT or CD62L^-/-^ memory P14 CD8^+^ T cells were transferred into naïve B6 mice and infected with LCMV. On day 50 post-infection, expression of (A,B) CD127 and KLRG1 or (C,D) CD27 and KLRG1 was analyzed on Thy1.1 memory P14 CD8^+^ T cells isolated from the blood. (E,F) WT and CD62L^-/-^ memory P14 CD8^+^ T cells were stimulated with GP_33-41_ peptide for 5 hours and expression of IFNγ was analyzed by intracellular stain.
(TIF)

S3 Fig Phenotype and function of WT and CD62L^-/-^ memory CD8^+^ T cells in the skin following the resolution of VacV infection. (A) Expression of CD69, core 2 O-glycans (identified with the monoclonal antibody 1B11), and CD44 on WT memory P14 CD8^+^ T cells isolated from the skin or spleen on day 40 after VacV-GP33 skin infection. (B,C) Quantification of (B) core 2 O-glycan expression (1B11) and (C) CD44 expression on both WT and CD62L^-/-^ memory P14 CD8^+^ T cells as shown in (A). (D) Expression of CD122 on memory P14 CD8^+^ T cells isolated from the spleen or skin as in (A). (E) Quantification of CD122 expression on both WT and CD62L^-/-^ memory P14 CD8^+^ T cells. (F) Memory P14 CD8^+^ T cells isolated from the skin on day 40 after VacV-GP33 skin infection were stimulated overnight with GP_33-41_ peptide and IFNγ expression was analyzed by intracellular stain. (G) Quantification of (F). (H) Surface expression of CD107a/b following overnight stimulation with GP_33-41_ peptide.
(TIF)

S4 Fig CD69+ T_RM_ CD8^+^ T cells in the skin generated by circulating memory CD8^+^ T cells are protected from IV labeling. (A) Experimental design to establish memory CD8^+^ T cells in the skin and to identify circulating memory CD8^+^ T cells using intravenous (IV) labeling. (B) Representative example of memory P14 CD8^+^ T cells in the blood and skin that were IV labeled following injection of CD8β antibody (C) Quantification of the percent of memory P14 CD8^+^ T cells in the skin that were IV labeled with CD8β antibody.
(TIF)

S5 Fig CD69+ T_RM_ in the skin generated by circulating memory CD8^+^ T cells are protected from antibody-mediated depletion. (A) Experimental design to determine if CD69+ memory CD8^+^ T cells in the skin generated from circulating memory CD8^+^ T cells are protected from antibody-mediated depletion. (B) Circulating frequencies of memory P14 CD8^+^ T cells prior to antibody administration. (C) Mice from (B) were administered control IgG or Thy1.1-depleting antibodies as described in *Materials and Methods*. Shown are representative FACS plots of memory P14 CD8^+^ T cells in the blood, spleen, or skin. (D) Quantification of memory P14 CD8^+^ T cells in the spleen and (E) skin following administration of control rat IgG or Thy1.1-depleting antibody.
(TIF)

S6 Fig Previously naïve CD8^+^ T cells express more CD103 than memory CD8^+^ T cells when stimulated with TGF-β following acute LCMV infection. (A) Naïve (Thy1.1/1.2) P14 CD8^+^ T cells were transferred with 1 x 10^5^ T_CM_ or T_EM_ P14 CD8^+^ T cells (Thy1.1/1.1) into B6 mice and subsequently infected with LCMV. On day 5 post-infection, splenocytes from infected mice were stimulated with 1 ng/ml TGF-β1 for 48 hours and expression of CD103 was analyzed. (B) Same experimental design as (A) except CD103 expression was quantified following stimulation with the indicated concentration of TGF-β1.
(TIF)

S7 Fig Memory CD8^+^ T cells express granzyme B after entering VacV-infected skin, but do not require CD62L to provide protection. (A) Naïve P14 CD8^+^ T cells were transferred into B6 mice and infected with LCMV. On day 60 post-infection, mice were challenged with VacV-GP33 on the left ear skin. On day 4 post-infection, expression of granzyme B by memory P14 CD8^+^ T cells in the skin, draining lymph node, and non-draining lymph node was analyzed by intracellular stain. (B) WT and CD62L^-/-^ B6 mice were infected with LCMV. On day 90 post-infection, LCMV-immune or naïve controls were infected with VacV-GP33 on the left ear skin and H2-D^b^-GP_33-41_-specific CD8^+^ T cells were identified in the blood. (C) Quantification of (B). (D,E) Same experimental design as (B,C) except viral load in the skin was quantified on (D) day 4 and (E) day 7 post-infection.
(TIF)
