# Supplementary figures and images for: Central memory CD8+ T cells become CD69+ tissue-residents during viral skin infection independent of CD62L-mediated lymph node surveillance
Source: PLoS Pathog. 2019 Mar 15;15(3):e1007633. doi: 10.1371/journal.ppat.1007633 (PMC6420010; doi:10.1371/journal.ppat.1007633)

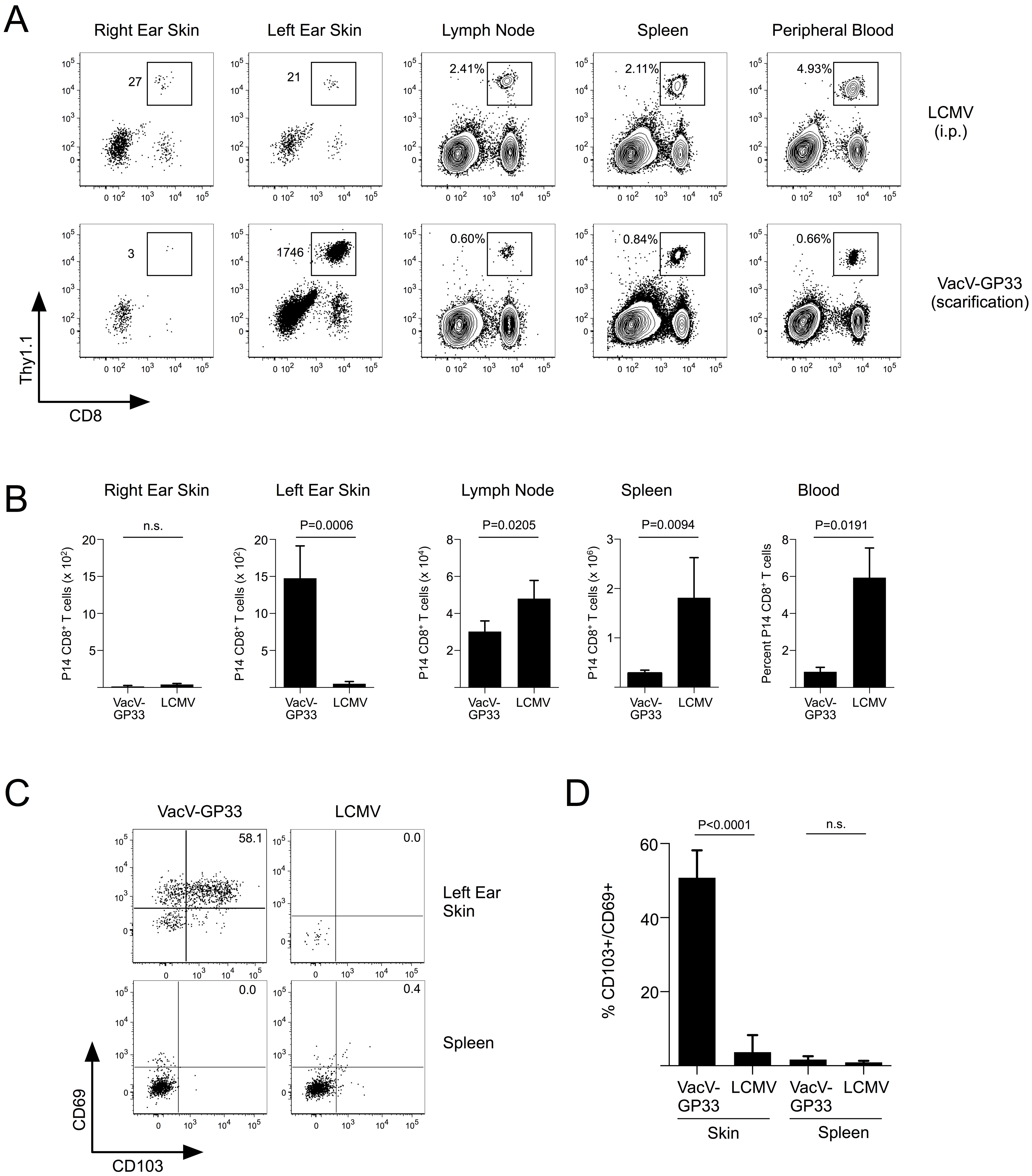

Supplement: S1 Fig — (A) 1 x 10^4^ naïve Thy1.1 P14 CD8+ T cells were transferred into naïve B6 mice which were then infected with either VacV-GP33 on the left ear skin or LCMV by i.p. injection. On day 40 post-infection, the distribution of memory P14 CD8+ T cells was analyzed in the indicated tissues. (B) Quantification of (A). (C) Expression of CD69 and CD103 of memory P14 CD8+ T cells from either the skin or spleen on day 40 post-infection. (D) Quantification of (C). (TIF) [file ppat.1007633.s001.tif]

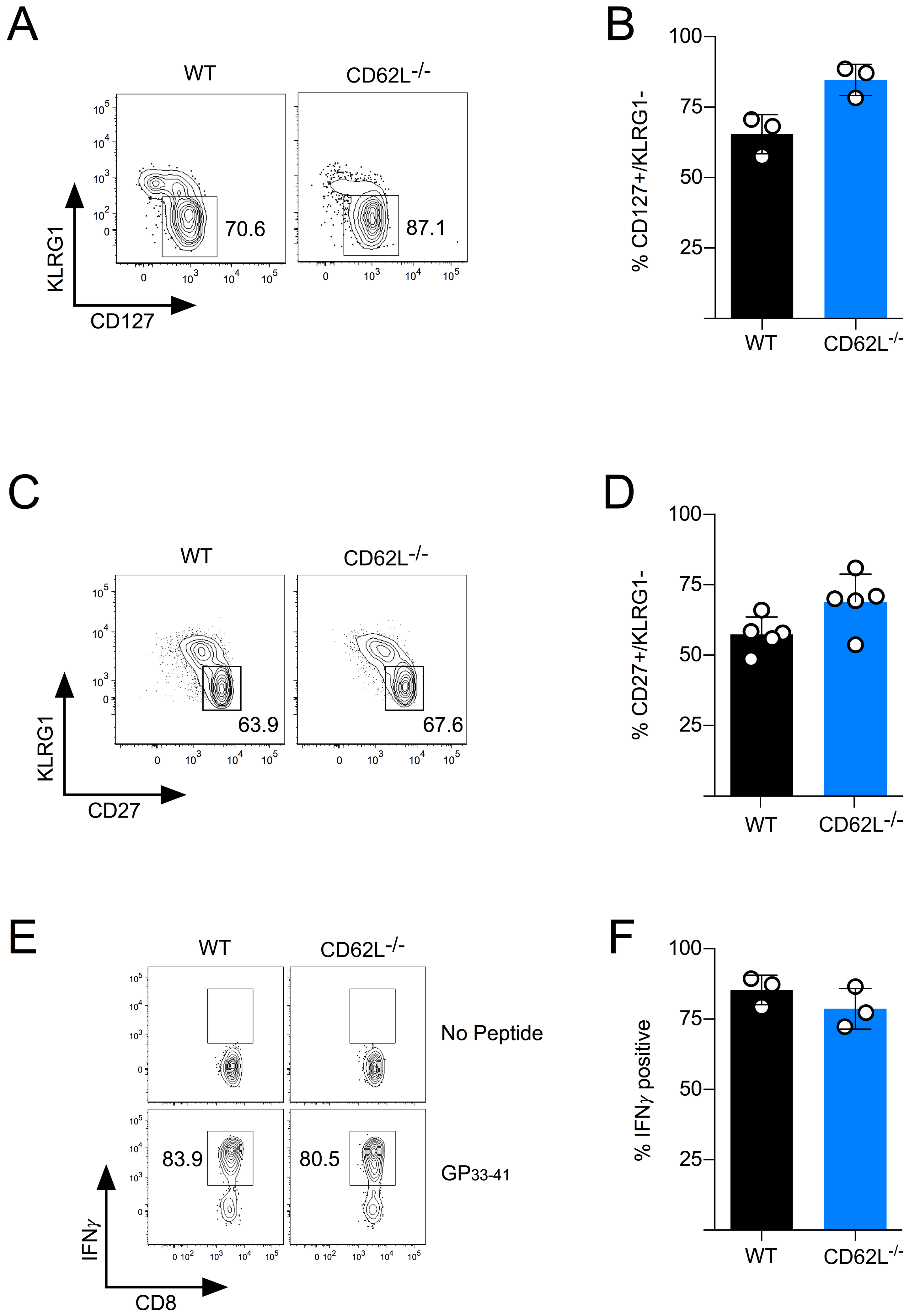

Supplement: S2 Fig — Naïve WT or CD62L-/- memory P14 CD8+ T cells were transferred into naïve B6 mice and infected with LCMV. On day 50 post-infection, expression of (A,B) CD127 and KLRG1 or (C,D) CD27 and KLRG1 was analyzed on Thy1.1 memory P14 CD8+ T cells isolated from the blood. (E,F) WT and CD62L-/- memory P14 CD8+ T cells were stimulated with GP33-41 peptide for 5 hours and expression of IFNγ was analyzed by intracellular stain. (TIF) [file ppat.1007633.s002.tif]

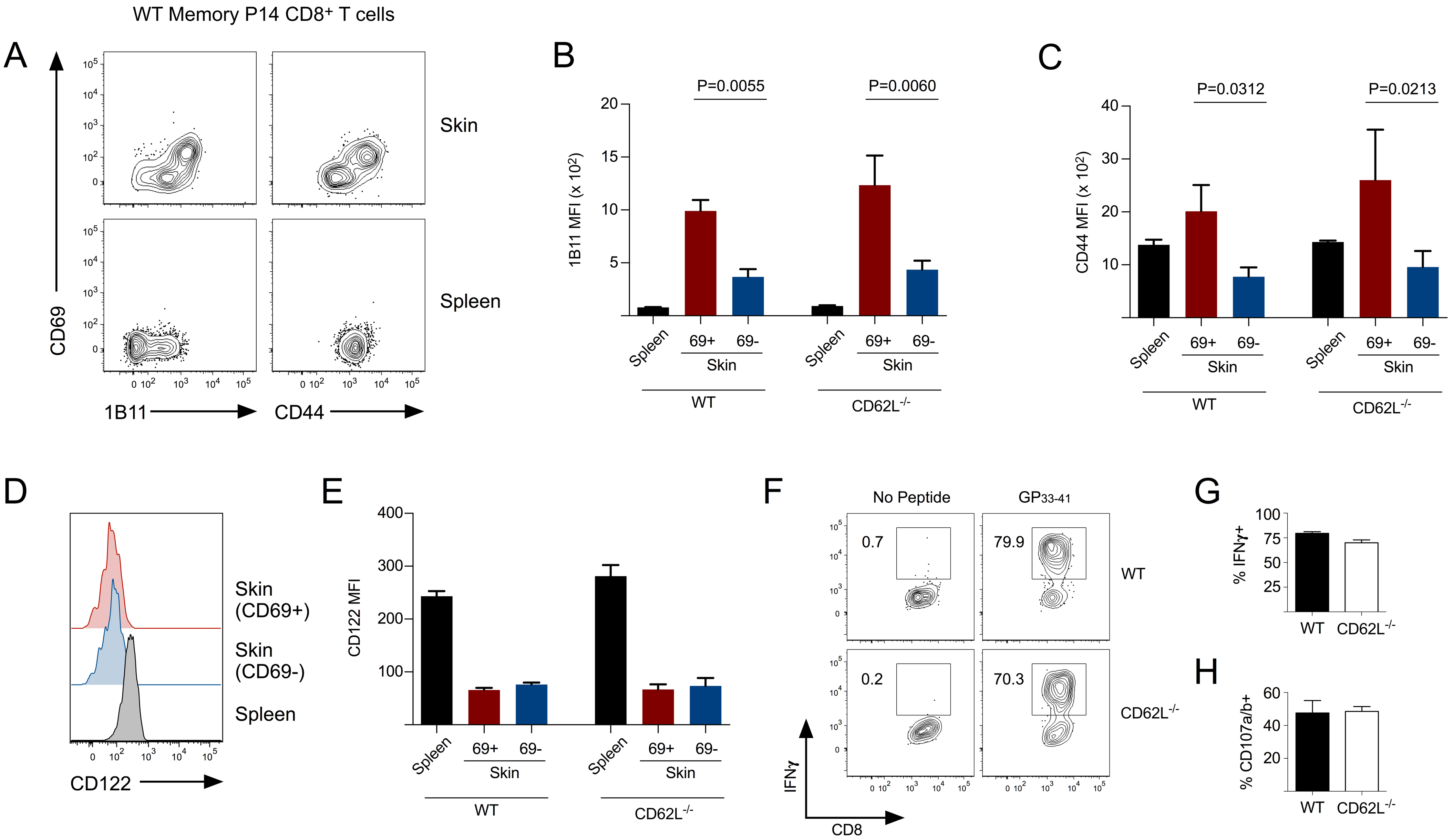

Supplement: S3 Fig — (A) Expression of CD69, core 2 O-glycans (identified with the monoclonal antibody 1B11), and CD44 on WT memory P14 CD8+ T cells isolated from the skin or spleen on day 40 after VacV-GP33 skin infection. (B,C) Quantification of (B) core 2 O-glycan expression (1B11) and (C) CD44 expression on both WT and CD62L-/- memory P14 CD8+ T cells as shown in (A). (D) Expression of CD122 on memory P14 CD8+ T cells isolated from the spleen or skin as in (A). (E) Quantification of CD122 expression on both WT and CD62L-/- memory P14 CD8+ T cells. (F) Memory P14 CD8+ T cells isolated from the skin on day 40 after VacV-GP33 skin infection were stimulated overnight with GP33-41 peptide and IFNγ expression was analyzed by intracellular stain. (G) Quantification of (F). (H) Surface expression of CD107a/b following overnight stimulation with GP33-41 peptide. (TIF) [file ppat.1007633.s003.tif]

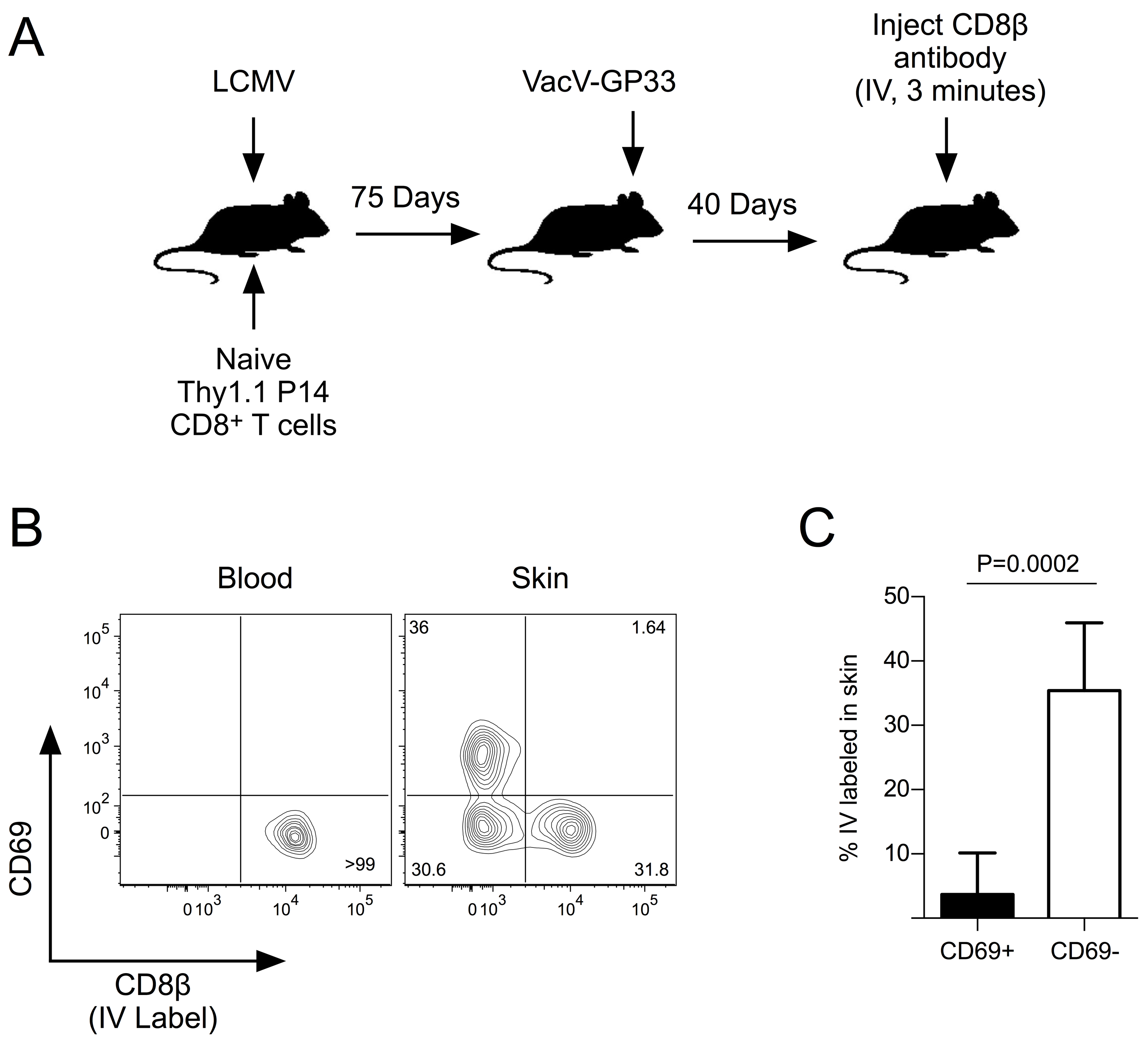

Supplement: S4 Fig — (A) Experimental design to establish memory CD8+ T cells in the skin and to identify circulating memory CD8+ T cells using intravenous (IV) labeling. (B) Representative example of memory P14 CD8+ T cells in the blood and skin that were IV labeled following injection of CD8β antibody (C) Quantification of the percent of memory P14 CD8+ T cells in the skin that were IV labeled with CD8β antibody. (TIF) [file ppat.1007633.s004.tif]

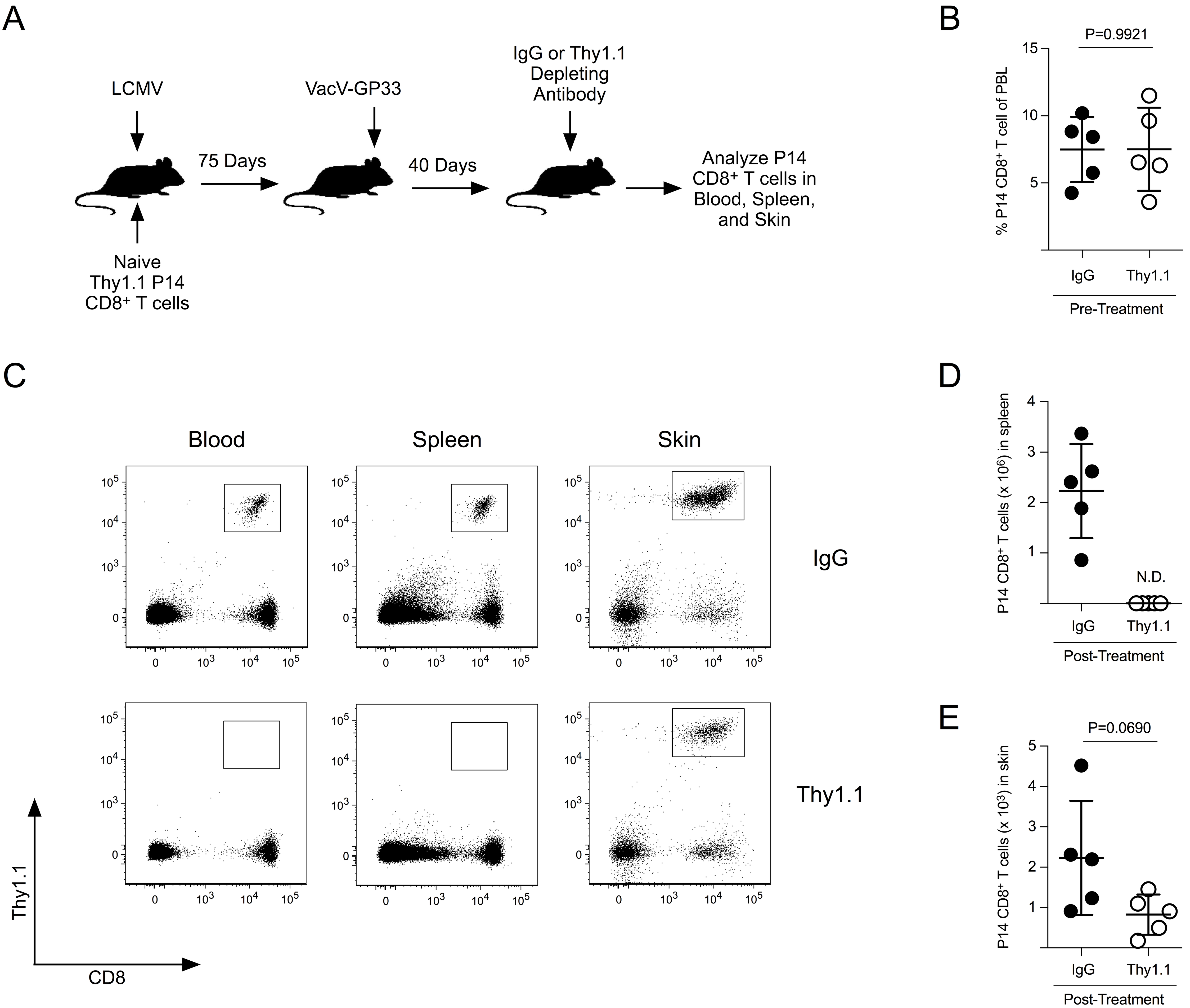

Supplement: S5 Fig — (A) Experimental design to determine if CD69+ memory CD8+ T cells in the skin generated from circulating memory CD8+ T cells are protected from antibody-mediated depletion. (B) Circulating frequencies of memory P14 CD8+ T cells prior to antibody administration. (C) Mice from (B) were administered control IgG or Thy1.1-depleting antibodies as described in Materials and Methods. Shown are representative FACS plots of memory P14 CD8+ T cells in the blood, spleen, or skin. (D) Quantification of memory P14 CD8+ T cells in the spleen and (E) skin following administration of control rat IgG or Thy1.1-depleting antibody. (TIF) [file ppat.1007633.s005.tif]

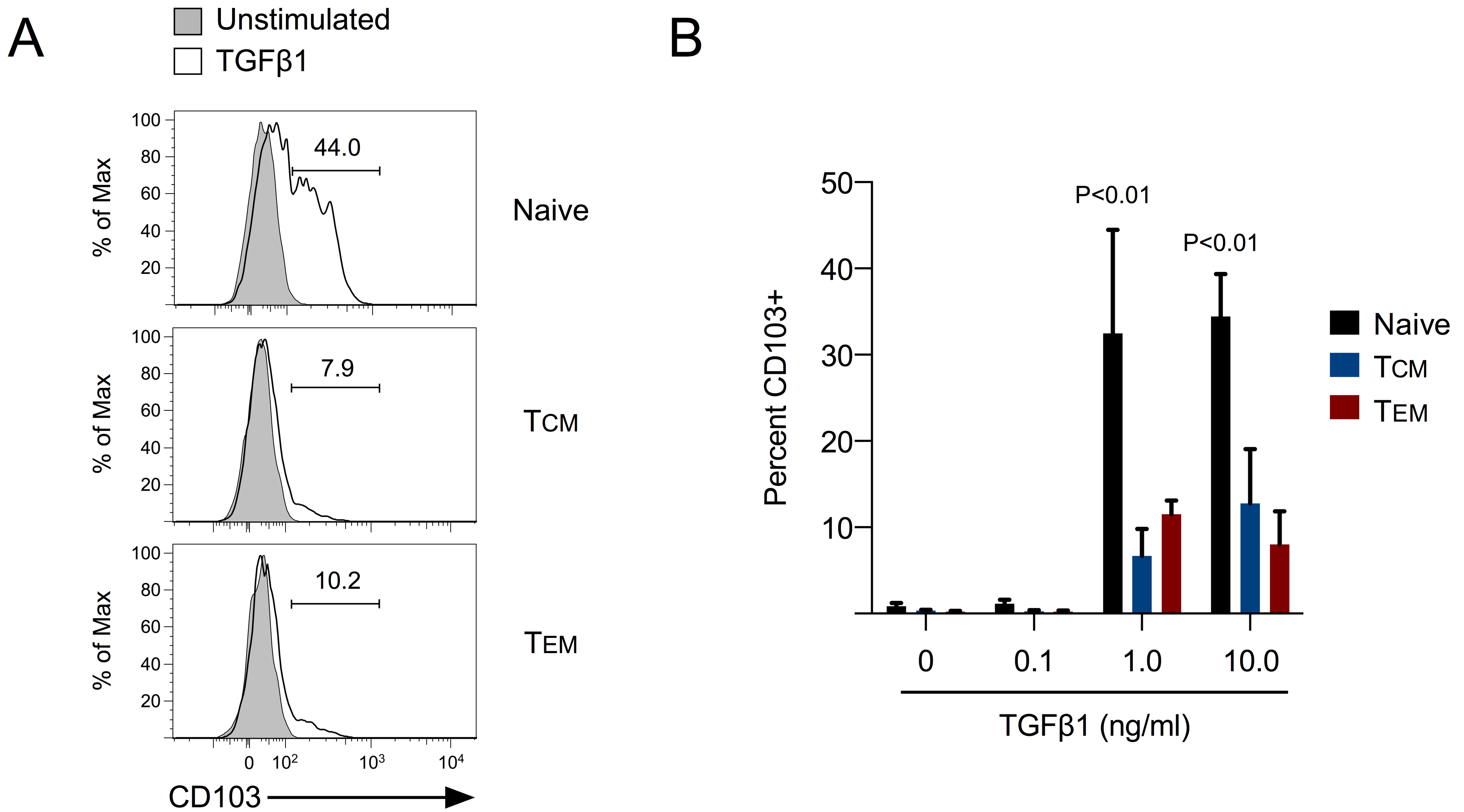

Supplement: S6 Fig — (A) Naïve (Thy1.1/1.2) P14 CD8+ T cells were transferred with 1 x 10^5^ TCM or TEM P14 CD8+ T cells (Thy1.1/1.1) into B6 mice and subsequently infected with LCMV. On day 5 post-infection, splenocytes from infected mice were stimulated with 1 ng/ml TGF-β1 for 48 hours and expression of CD103 was analyzed. (B) Same experimental design as (A) except CD103 expression was quantified following stimulation with the indicated concentration of TGF-β1. (TIF) [file ppat.1007633.s006.tif]

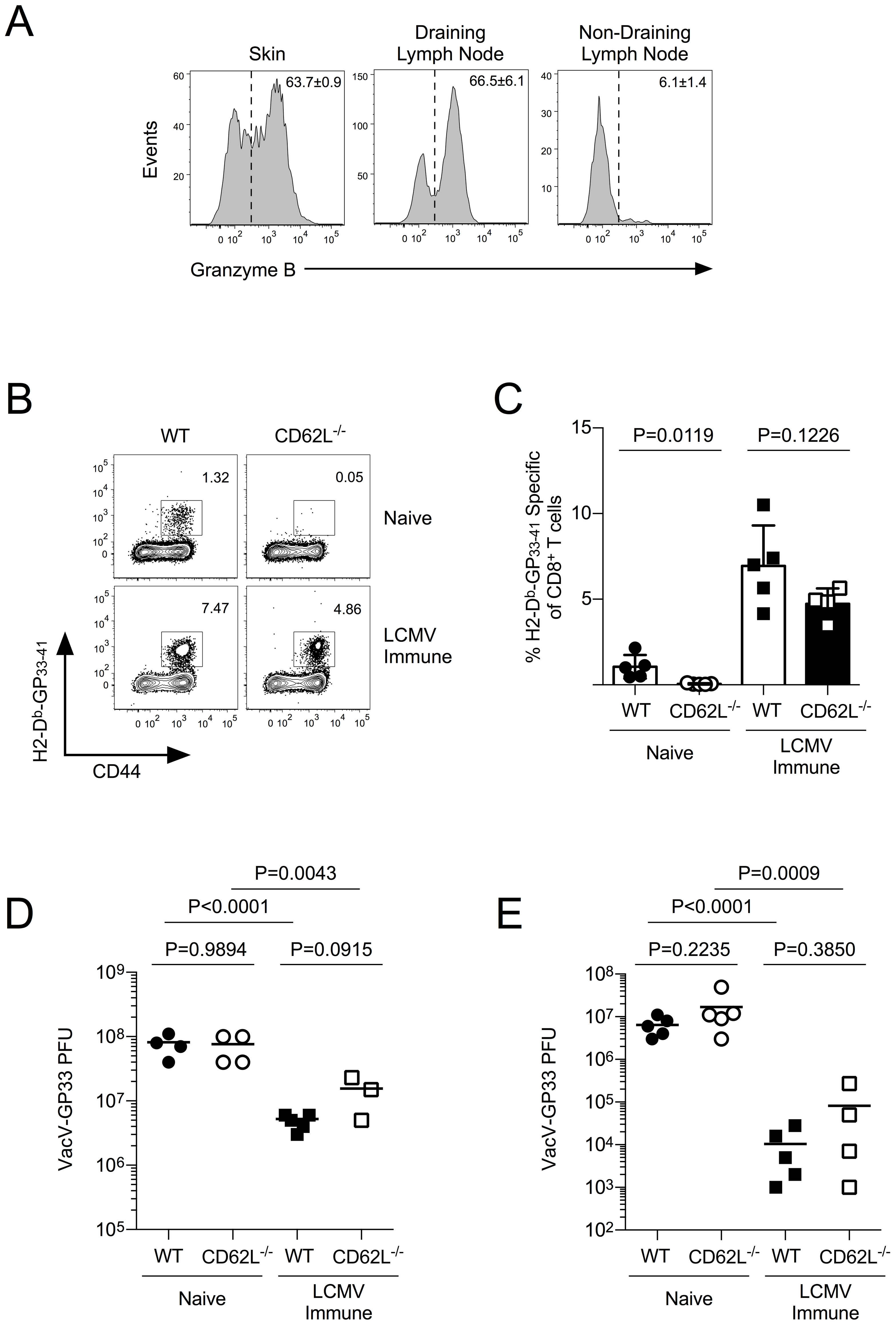

Supplement: S7 Fig — (A) Naïve P14 CD8+ T cells were transferred into B6 mice and infected with LCMV. On day 60 post-infection, mice were challenged with VacV-GP33 on the left ear skin. On day 4 post-infection, expression of granzyme B by memory P14 CD8+ T cells in the skin, draining lymph node, and non-draining lymph node was analyzed by intracellular stain. (B) WT and CD62L-/- B6 mice were infected with LCMV. On day 90 post-infection, LCMV-immune or naïve controls were infected with VacV-GP33 on the left ear skin and H2-Db-GP33-41-specific CD8+ T cells were identified in the blood. (C) Quantification of (B). (D,E) Same experimental design as (B,C) except viral load in the skin was quantified on (D) day 4 and (E) day 7 post-infection. (TIF) [file ppat.1007633.s007.tif]
